# New Complexes of Antimony(III) with Tridentate *O*,*E*,*O*-Ligands (E = O, S, Se, Te, NH, NMe) Derived from *N*-Methyldiethanolamine

**DOI:** 10.3390/molecules28134959

**Published:** 2023-06-24

**Authors:** Uwe Böhme, Marcus Herbig

**Affiliations:** Institut für Anorganische Chemie, TU Bergakademie Freiberg, Leipziger Str. 29, 09599 Freiberg, Germany; uwe.boehme@chemie.tu-freiberg.de

**Keywords:** antimony complexes, molecular structure, quantum-chemical analysis

## Abstract

We synthesized a series of new antimony(III) compounds by reaction of Sb(OEt)_3_ with organic ligands of the type E(CH_2_-CH_2_-OH)_2_, with E = NH, NMe, O, S, Se, and Te. The synthesized compounds have the general composition [E(CH_2_-CH_2_-O)_2_]Sb(OEt). For comparison, the compound (O-CH_2_-CH_2_-S)Sb(OEt) was prepared. All compounds are characterized using NMR, IR, and Raman spectroscopy. The molecular structures of the products reveal the formation of chelate complexes, wherein the ligand molecules coordinate as tridentate *O*,*E*,*O*-ligands to the antimony atom. Dimer formation in the solid state allows the antimony atoms to reach pentacoordination. Quantum chemical calculations including topological analysis of electron density reveal that there are polar shared bonds between antimony and the oxygen atoms bound to antimony. The interactions between the donor atom E and the Sb atom and the interactions in the dimers can be characterized as Van der Waals interactions. The reactivity of [MeN(CH_2_-CH_2_-O)_2_]Sb(OEt) was investigated as an example. For this purpose, the compound reacted with a range of organic compounds such as carboxylic acids and carboxylic anhydrides and small molecules like CO_2_ and NH_3_. This study establishes a new and easy accessible class of antimony(III) compounds, provides new insights into the chemistry of antimony compounds and opens up new opportunities for further research in this field.

## 1. Introduction

New main group metal complexes for catalysis have been the subject of increasing research recently due to their good availability and low cost. For example, early main group metals can catalyse many reactions like polymerisation, hydrofunctionalisation and cross-coupling reactions [1]. Heavier pnictogens offers new possibilities of catalytic applications because there are multiple stable oxidation states (usually at least 0, +3 and +5). Additionally, Arduengo and Stewart reported low valent pnictogen complexes of phosphorous to bismuth [2,3]. In the group of pnictogens, the tendency for hybridization lowers with higher atomic number. So the lone pair of pnictogen (III) compounds gains a more s-orbital character and therefore, the lower oxidation states become preferable and the pnictogen compounds become more oxidizing with an increasing atomic number [4]. Furthermore, the heavier pnictogens show a Lewis acidity based on the σ* orbitals. This orbital can be considered as isolobal to the vacant p-orbital in boranes for antimony compounds [5]. The Lewis acidity of antimony compounds can be increased by the introduction of electron withdrawing ligands [6]. So, Lewis acidic antimony compounds are used as fluorine sensors [7,8,9] and catalysts [10,11,12,13]. The redox chemistry between antimony (III) and (V) complexes opens up further possibilities in catalysis [14].

Besides their applications as sensor materials or catalysts, antimony compounds have been used for the treatment of the parasite disease leishmaniasis for more than half a century [15]. Recently, antimony complexes have been investigated for different applications: antimony(V) complexes were tested for their cytotoxic activity [16], for their antibacterial activities and cytotoxicity against macrophages [17]. Different sugars were combined with antimony(V) complexes to modulate the oral bioavailability of antimony containing drugs [18]. The biological potential of antimony(III) dithiocarbamate complexes has been recently reflected in a review article [19]. Antimony(III) and (V) phthalocyanine derivatives absorb near infrared light, which makes these compound attractive as potential photosensitizers for photodynamic cancer therapy and as an infrared filter for plasma display and silicon photodiodes [20].

Diethanolamine is a widely used compound bearing two electron withdrawing hydroxy moieties and a nitrogen donor atom. Complexes of transition metals with diethanolamine and related ligand molecules have been investigated for decades [21,22,23,24]. Also, complexes of diethanolamine with tin have been reported [25] and silatranes were studied extensively, e.g., [26,27,28,29]. Nonetheless, only a few antimony compounds of this type have been reported so far [30,31,32,33].

Herein, we present new antimony complexes with bi- and tridentate ligands of the diethanolamine class with different chalcogens and nitrogen as donor atoms.

## 2. Results and Discussion

**Figure 1:**
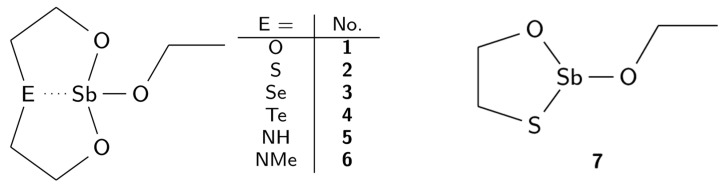
Synthesized compounds.

To investigate new antimony complexes with bi- or tridentate ligands, we synthesized the compounds shown in Figure 1. The ligand system was chosen to be as flexible as possible. The ligand system is similar to pincer ligands, but has two (formally) covalent bonds to the central atom (Sb). Compounds **1** to **6** contain an eight-membered ring with a donor atom E in the ligands backbone, while compound **7** is for comparison without this additional donor atom. The donor atoms include all stable chalcogens and nitrogen. So, the influence of the nature of the donor to the antimony atom can be investigated.

### 2.1. Syntheses

Except for the ligands of **3** and **4**, all ligands are commercially available. The selenium and tellurium ligands were synthesized using a literature protocol [34].

Many organometallic compounds are synthesized starting with the element chlorides. Since antimony(III)-chloride is a strongly hygroscopic and soft solid (trivial name: butter of antimony) [35], it is transformed into other starting materials as antimony(III)-acetat [Sb(OAc)_3_], tris(dimethylamino)antimony [Sb(NMe_2_)_3_] or triethoxyantimony [Sb(OEt)_3_]. The latter two are colourless liquids that can be effortlessly handled with the standard Schlenk technique. For the synthesis of new complexes with three Sb-O bonds, Sb(OEt)_3_ is an excellent starting material. Ethanol is formed as joint product in the reactions of this starting material with the ligands. The reactions were carried out in cyclohexane, which forms an azeotrope with ethanol boiling at 68 °C. Therefore, the reaction progress was monitored by the temperature at still head. If the temperature rises over 70 °C to 80 °C (boiling point of cyclohexane), the product has been formed in a quantitative yield. After the removal of the solvents under reduced pressure, the crude products were purified by recrystallization from chloroform to remove excess ligands. A similar synthesis procedure was used by Gupta et al. [30].

For temperature sensitive ligands (Se and Te derivatives), another synthesis procedure has been developed: The ligand and Sb(OEt)_3_ were dissolved together in absolute ethanol and the products crystallized from this solution after several hours. Since the complexes are slightly soluble in ethanol, the yield of this synthesis protocol is lower, but the purity is higher.

### 2.2. NMR Data

The NMR spectra of the ligands show the signals as expected, but the spectra of the products **1**–**7** are more complicated. In the ^1^H NMR spectra of **2**, for example (see Figure S3 in the Supplementary Materials), there are four broad signals for the protons of the tridentate ligand. This indicates that the ring system is not flexible and the equatorial and axial proton positions become non-equivalent. This is a first hint to the coordination of the inner donor atom to antimony. Additionally, the proximity to the quadrupole Sb atom broadens the signals. In the ^13^C NMR spectrum, the signal of the inner-cyclic O-CH_2_ at 58.0 ppm becomes less intense and broad because of the proximity to the Sb atom (see Figure S4 in the Supplementary Materials).

### 2.3. Vibrational Spectroscopy

Since there are only a few vibration frequencies for common covalent trivalent antimony compounds reported in the literature [36,37,38], the vibrational frequencies are calculated using quantum mechanical methods. As an example, the recorded and the calculated IR and Raman spectra of **1** are shown in Figure S5 in the Supplementary Materials. A comparison of the infrared spectra of the free ligand *N*-methyldiethanolamine and the antimony complex **6** are shown in Figure S6 in the Supplementary Materials. It is obvious that the ν(O-H) above 3000 cm^−1^ in the ligand cannot be found in the complex. Except for this region, the IR spectra of the ligand *N*-methyldiethanolamine and the complex **6** look quite similar, since most of the intense vibrations are caused by the light atoms. For a better understanding of the vibrations, including the antimony atom, Raman spectra were used.

According to the quantum chemical calculations, all complexes show characteristic bands at about 600 cm^−1^, which can be identified as ν(Sb-OEt). There are two bands from 450 to 500 cm^−1^, which are the symmetric and asymmetric inner-cyclic ν(Sb-O). The band around 1075 cm^−1^ arises from the inner-cyclic ν(C-O). A ν(Sb-E) can be found in the calculated spectra around 150 to 70 cm^−1^.

The calculations predicted a ν(Sb…E) with a decreasing wave number and an increasing atomic number of E. These bands can be found in the Raman spectra with the same trend. The presence of the Sb-OEt moiety is also confirmed by the Raman spectra, since there are bands around 600 cm^−1^ (see Table 1).

### 2.4. Molecule Structures

**Figure 2:**
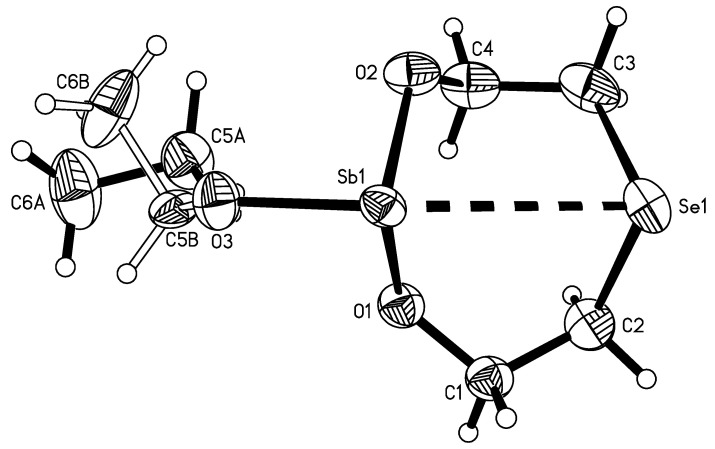
Molecule structure of **3** with atomic numbering scheme, shown with 50% probability ellipsoids (The same atomic numbering was applied in the crystal structures of **1**, **2**, and **4**).

**Figure 3:**
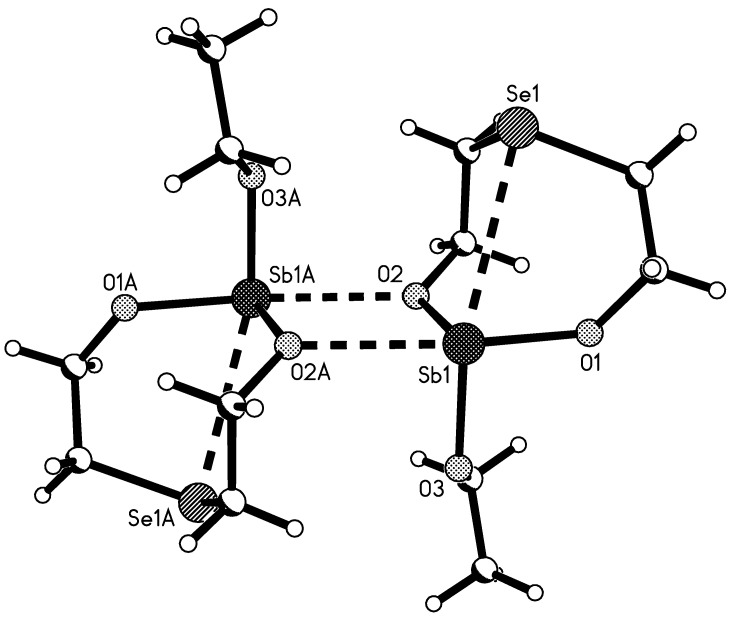
Dimer formation in the crystal structure of **3**. The disorder at the ethyl group was omitted for clarity (Symmetry equivalent atoms are generated by symmetry operation 1 − x, 1 − y, 1 − z).

The molecule structures of compounds **1**–**4**, **6** and **7** were determined by single crystal X-ray diffraction. The compounds **1**–**3** crystallize in the triclinic space group P-1 as isostructural compounds. Compound **4** crystallizes in the monoclinic space group I2/a. Compound **6** crystallizes with one molecule chloroform in the asymmetric unit in the triclinic space group P-1, which is not isomorphous with **1**–**3**. Compound **7** crystallizes in the monoclinic space group P2_1_/n. The basic geometric properties of the compounds **1**–**4**, and **6** are explained using compound **3** as an example. One ethoxy group is bound to the antimony atom. The ethoxy group is disordered in the crystal structures of **2** and **3**, and the details are described in the experimental part. The tridentate chelate ligand binds via the lateral oxygen atoms and the central donor atom (Se in compound **3**) to the antimony atom. This leads formally to a tetracoordinate antimony atom (Figure 2). The antimony atom is coordinatively unsaturated and forms dimers in the solid state (Figure 3). The dimer is generated by an inversion centre. One oxygen atom (O2) from a neighbouring molecule, coordinates at the antimony atom. The distance Sb1…O2A is 2.580(2) Å in **3**.

The intermolecular interaction between two molecules lead to a quadrilateral arrangement of the atoms O2-Sb1…O2A-Sb1A. The atoms are not arranged as a rectangle, but as a parallelogram with the antimony atoms in the centres of the acute angles and the oxygen atoms in the centres of the obtuse angles. For compound **3**, these angles are 66.08(8)° for O2-Sb1-O2A and 113.92(8)° for Sb1-O2-Sb1A. Similar values are found for the other dimers of the compounds **1**, **2**, **4**, and **6**. The dimer formation leads formally to pentacoordinate antimony atoms. However, one should consider the presence of a free electron pair at the antimony(III) atoms. This supplements the coordination geometry of the antimony atoms and delivers an explanation for the arrangement of the dimer as a parallelogram. The free electron pairs simply need “space” at the periphery of the dimers. The orientation of the electron pair will be discussed in more detail in the quantum chemical section below.

The bond lengths Sb-O in compound **3** are 1.997(2) and 2.008(2) Å for Sb1-O1 and Sb1-O2, respectively. The bond Sb1-O3 is 1.966(2) Å. Similar values are also found in the other molecule structures. The bond to the ethoxy group is always shorter than the bonds to the oxygen atoms from the chelate ligand. These bond lengths agree well with the sum of covalent radii for Sb and O (2.07 Å).

**Figure 4:**
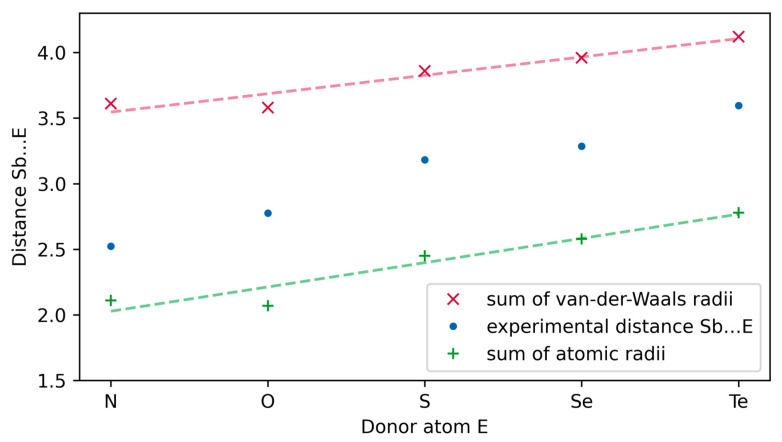
Distances Sb…E for compounds **1**–**4**, and **6**, sum of atomic radii and sum of Van der Waals radii (all values in Å). The dashed lines illustrate the trends.

The coordination of the central donor atom E is interesting and deserves closer examination. For this purpose, the data for the atomic distances Sb…E have been compiled in Table 2. In addition, the sums of the atomic radii and the sums of the Van der Waals radii are listed there. Figure 4 provides a graphical illustration of these distances. As can be seen from Figure 4, the distances found in the molecule structures are shorter than the sum of Van der Waals radii for all compounds. The experimental found distances are longer than the sum of covalent radii. These two facts indicate the presence of a donor–acceptor interaction between antimony and the central donor atoms E.

**Figure 5:**
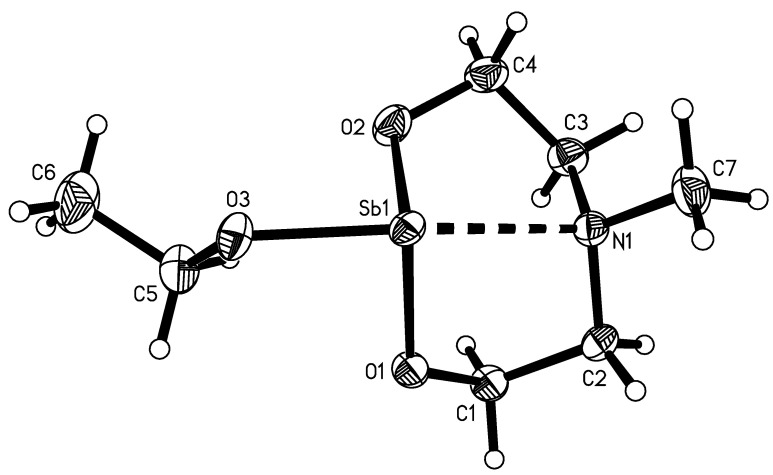
Molecule structure of **6** with atomic numbering scheme, shown with 50% probability ellipsoids.

The distance Sb…N in compound **6** is significantly shorter than one would expect in the series of these chemical compounds. This is probably due to the bonding conditions of the nitrogen atom. In contrast to the other donor atoms with E(CH_2_—)_2_ units, the nitrogen atom already has three bonding partners within a N(Me)(CH_2_—)_2_ unit and additionally exercises the donor function to the antimony atom (Figure 5). If we compare the bond angles C2-E-C3 in the series of molecular structures (Table 3), we find that the elements of nitrogen and oxygen from the second period of the periodic table with angles around 113° are clearly more obtuse than the angles for the elements of the higher periods (99.43 to 102.4°).

**Figure 6:**
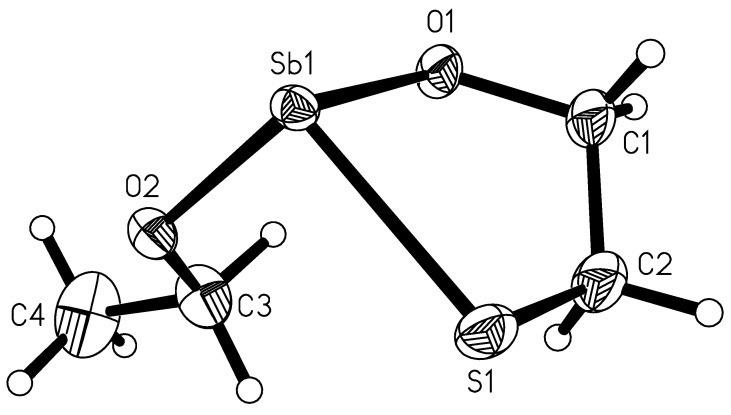
Molecule structure of **7** with atomic numbering scheme, shown with 50% probability ellipsoids.

**Figure 7:**
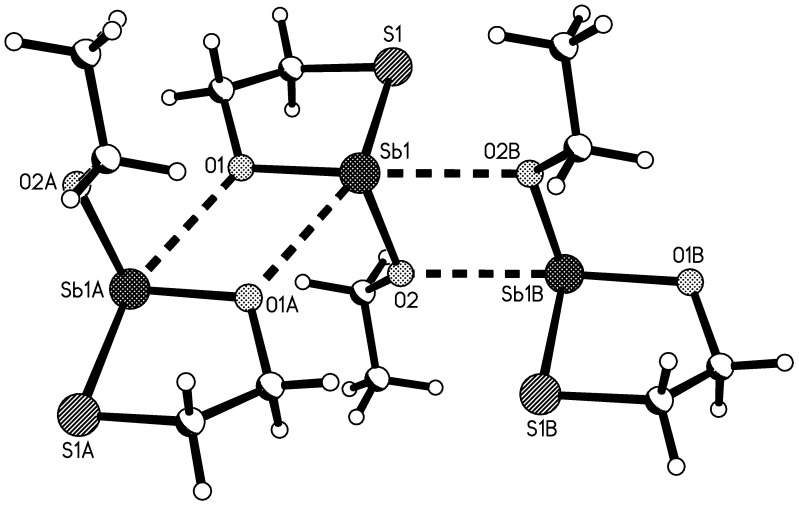
Intermolecular interactions between oxygen and antimony in the crystal structure of **7** (Symmetry equivalent molecule A is generated by symmetry operation 1 − x, 1 − y, 1 − z. Symmetry equivalent molecule B is generated by symmetry operation 1 − x, 2 − y, 1 − z).

The crystal structure of compound **7** gives information about what happens if we replace the tridentate dianionic chelate ligand by a bidentate dianionic ligand. Molecule **7** consists of an antimony(III) ion bound to an ethoxy group and the dianionic ligand. The bond lengths Sb-O have similar values as in the other molecule structures (2.020(2) Å for Sb1-O1 and 1.991(3) Å for Sb1-O2). The Sb-S distance is 2.456(1) Å, which corresponds well with the sum of covalent radii (2.45 Å). The antimony atom is triple-coordinated (Figure 6). This leaves a lot of space for intermolecular interactions, which are found in the crystal structure between each antimony atom and two oxygen atoms from neighbouring molecules (Figure 7). The distances Sb1…O1A and Sb1…O2B are 2.579(3) and 2.563(3) Å, respectively. These interactions make the antimony atoms pentacoordinate. The ladder-type Sb…O contacts lead to infinite chains of molecules parallel to the crystallographic b-axis.

### 2.5. Quantum Chemical Analysis

For further investigations of the influence of the donor element E and the dimer formation in the crystal, QTAIM (quantum theory of atoms in molecules [41,42]) analysis was performed using the structures optimized at the B97-3c [43] level of theory as a starting point for the structure optimization with the PBE0 [44,45]/def2-TZVPP [46] level. The wave function calculations were made at PBE0/def2-TZVPP with the NOECP keyword to exclude any pseudo-potential.

**Figure 8:**
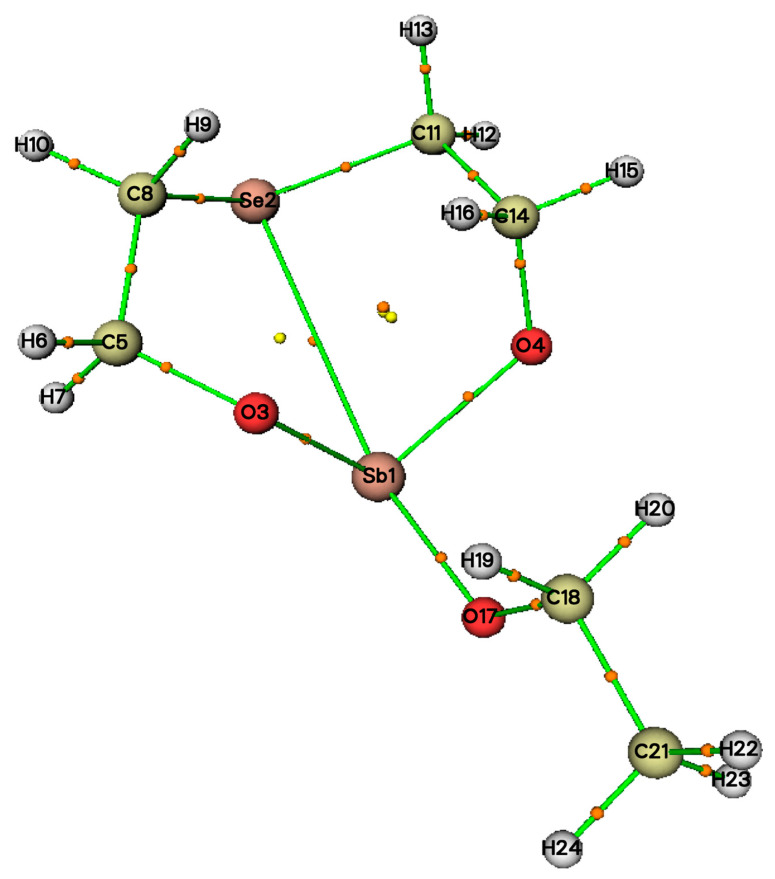
Graphical representation of the topological analysis of **3**. Bond critical points are marked in orange; ring critical points in yellow. The numbering of the atoms results from the line number of the input structure (see Supplementary Materials). (Remark: There is an additional BCP between H16 and O3 with a corresponding RCP in the Sb1-O4-C14-H16-O3 ring, which overlaps with the RCP of the Sb1-O4-C14-C11-Se2 ring in this figure).

**Figure 9:**
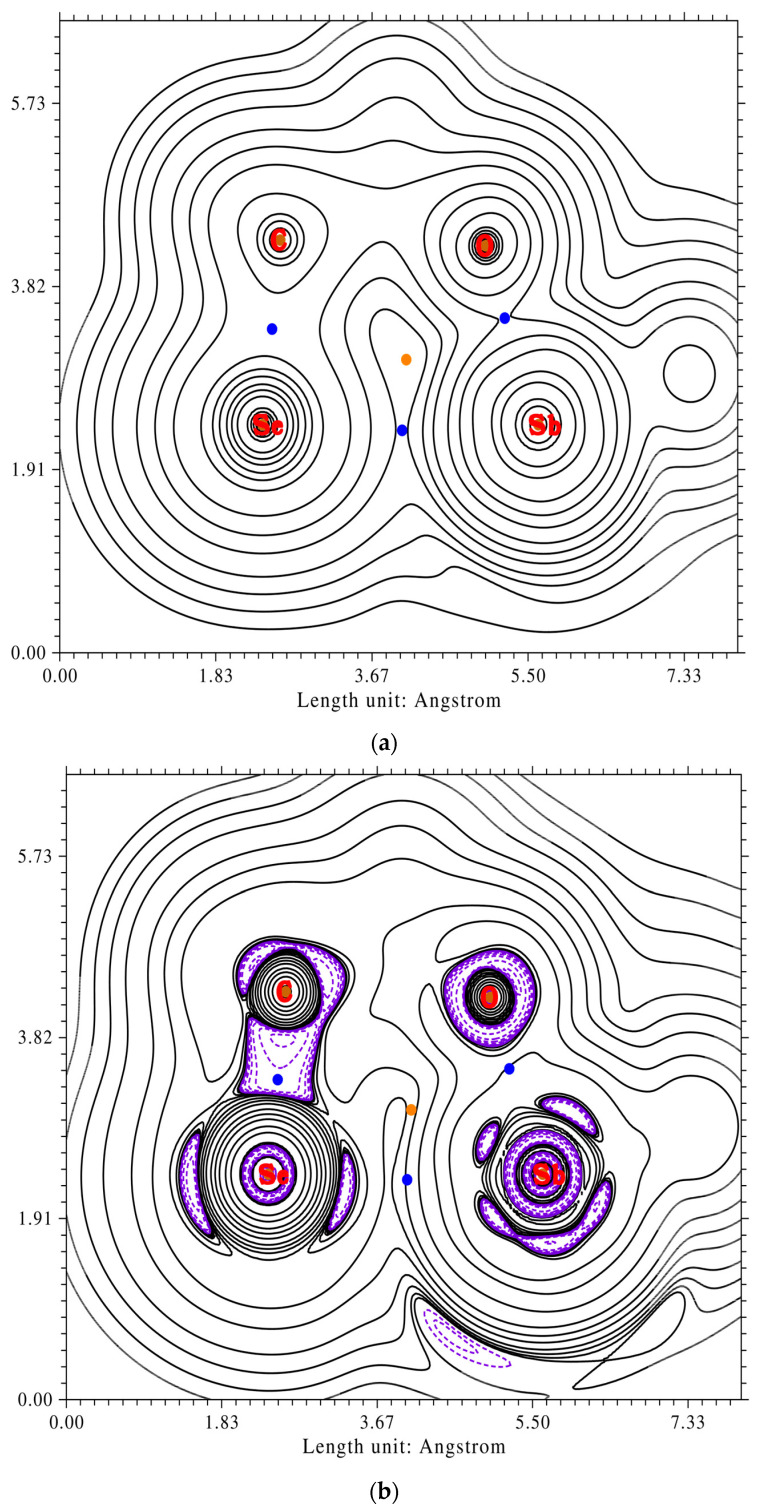
Electron density (**a**) and Laplacian of the electron density (**b**) of **3** in the Sb4-Se9-O7 plane. BCPs are marked in blue and RCPs in orange. Positive values of **∇**^2^ρ are drawn with solid lines and represent regions of charge depletion; negative values are drawn with purple dashed lines and represent regions of charge concentration. Contour values in atomic units are 0.001, 0.002, 0.004, 0.008, 0.02, 0.04, 0.08, 0.2, 0.4, 0.8, 2, 4, 8, 20, 40, 80, 200, 400, 800.

In all molecules, the Poincaré–Hopf relationship [41] is accomplished. There are bond critical points (BCPs) between Sb and the donor element E as well as two ring critical points (RCPs) inside the five-membered subrings of the chelate ligand-Sb ring system (see Figure 8). The topological data for **3** are shown as an example in Table 4. The data for all other compounds can be found in the Supplementary Materials. The electron density contour plot as well as a plot of the Laplacian of the electron density are shown in Figure 9.

The Sb-O bond is used for comparison with the Sb…E interactions. All BCPs between Sb and O have high electron densities and G/ρ is larger than 1 while H is negative. The graphical representation of the Laplacian (Figure 9) shows an electron depletion at the Sb atom in the direction of the O atom as well as an accumulation of charge at the O atom in the direction of the Sb atom. According to the criteria proposed by Macchi et al. [41,47], these bonds should be considered as polar shared bonds. The BCP between Sb and Se has a quite low electron density, G/ρ is nearly 1 and H > 0. The compounds **1**, **2**, **4**, **5,** and **6** feature values for G/ρ > 1 (see Supplementary Materials) and therefore all these Sb…E interactions can be characterized as closed-shell interactions (e.g., Van der Waals interactions). Furthermore, the graphical representation of the Laplacian of the electron density in Figure 9 shows the lone pairs of the Se atom as areas of valence charge concentration. One of the lone pairs shows into the direction of the Sb atom and on this atom the electron density is depleted in this direction, highlighting the nature of the interaction as Van der Waals or ionic interactions.

**Figure 10:**
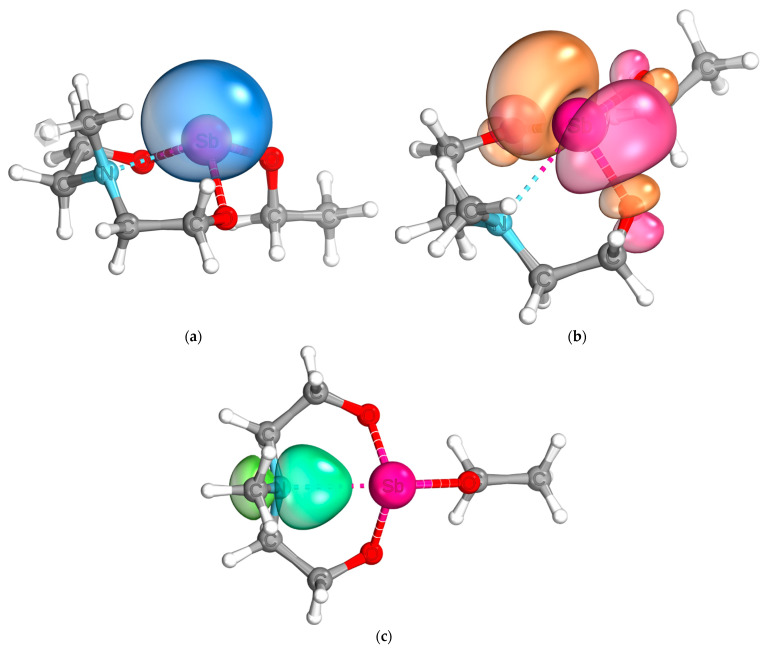
Sb-lone pair IBO (**a**), Intrinsic bond orbitals of the energetically lowest unoccupied IBO (**b**) and Sb…N interaction (**c**) in compound **6**.

In addition to the topological analysis, intrinsic bond orbitals (IBO [48]) were calculated at the same level of theory to illustrate the orbital interactions. The lowest unoccupied IBO, the lone pair of Sb, and the Sb…N interaction of compound **6** are shown in Figure 10 as representative examples.

As the IBOs show, the energetically lowest unoccupied IBO of the molecule is located mostly at the Sb atom. The lone pair of the Sb atom can be described as nearly spherical, meaning a high s-orbital character, as one would expect for a fifth period element [49]. The lone pair of the N atom is directed towards the Sb atom, indicating the Sb…N interaction in this molecule.

**Figure 11:**
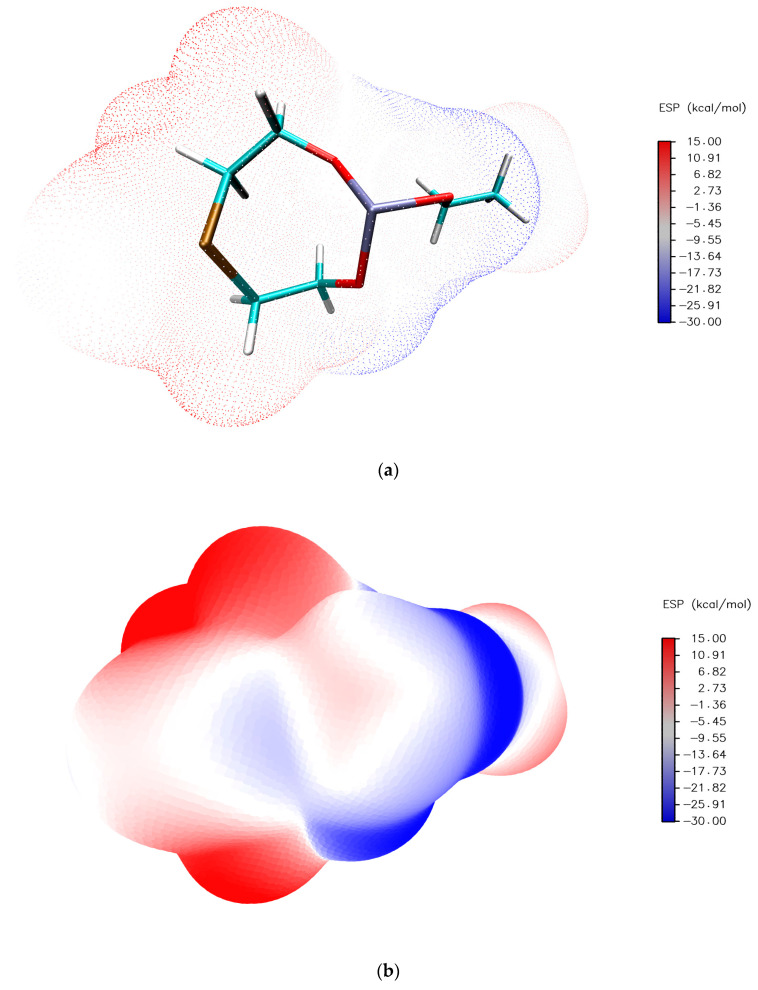
The molecule (**a**) and the electrostatic potential map (**b**) of **6**.

To investigate the σ-hole at the antimony atom in 6, an electrostatic potential map was created. As can be seen in Figure 11, there is a positive potential at the antinomy atom opposite the three oxygen substituents, which can be interpreted as weak σ-hole.

**Figure 12:**
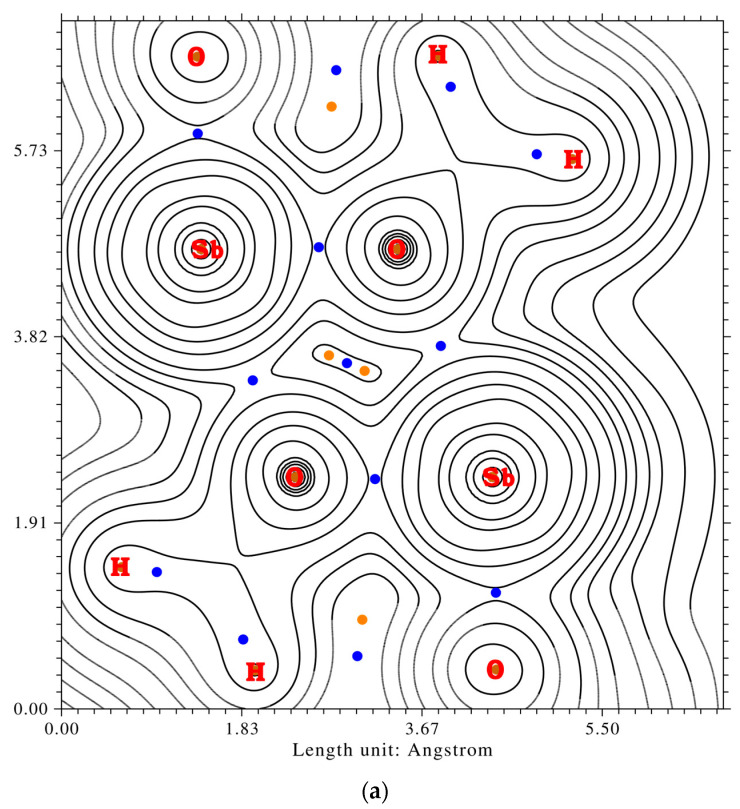

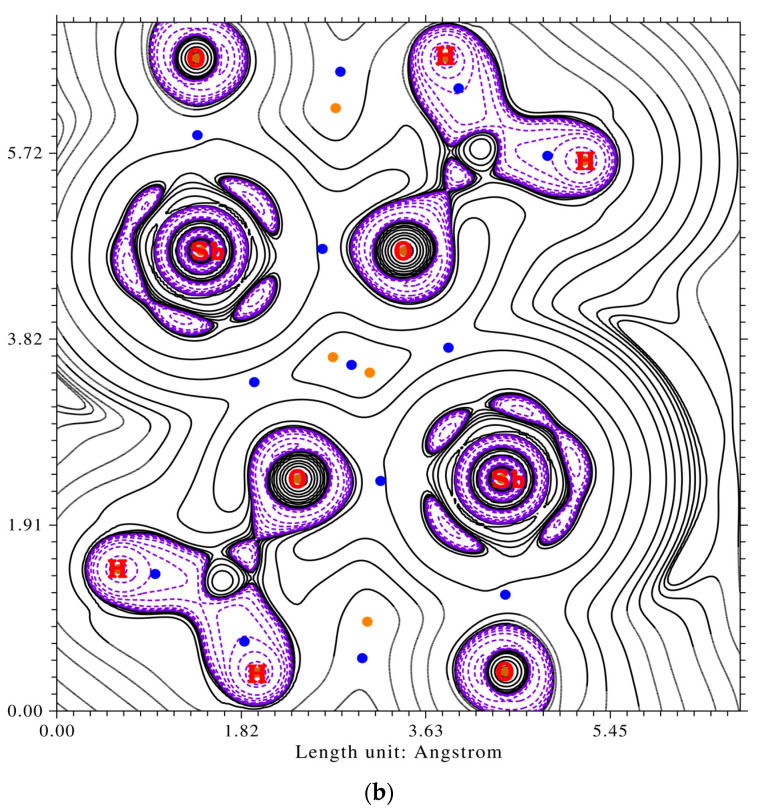
Electron density (**a**) and Laplacian of the electron density (**b**) of **3** dimers in the Sb-O-Sb-O-plane. BCPs are marked in blue and RCPs in orange. Positive values of ∇^2^ρ are drawn with solid lines and represent regions of charge depletion; negative values are drawn with purple dashed lines and represent regions of charge concentration. Contour values in atomic units are 0.001, 0.002, 0.004, 0.008, 0.02, 0.04, 0.08, 0.2, 0.4, 0.8, 2, 4, 8, 20, 40, 80, 200, 400, 800.

The formation of dimers in the solid state is another interesting aspect of these compounds. Further quantum chemical investigations were performed to gain insight into the underlying interactions. The solid-state structure of **3** was used as a starting point and all atom positions were optimized without restrictions. The topological analysis shows five BCPs inside the Sb-O-Sb-O-parallelogram. Two indicate the intramolecular Sb-O bonds, two intermolecular Sb…O interactions and one BCP is in the middle of the parallelogram. The central BCP has a very low electron density and is considered as weak contact. The graphical representation of the electron density and the Laplacian of the electron density can be found in Figure 12 and the numeric data in Table 5. Additionally, a weak O…H interaction is found between one O atom and a nearby CH moiety.

**Table 5:** Results of topological analysis of the Sb-O-Sb-O-plane in the dimeric structure of **3** (all values in atomic units). Parameters are charge density (ρ), its Laplacian (∇^2^ρ), kinetic energy density (G), total energy density (H), and electronic potential energy density (V). The atom numbering is the same as in Figure 13, resulting from the quantum chemical calculation file (see Supplementary Materials). Only the atom numbering of one molecule is shown for clarity, where applicable.

	ρ	∇^2^ρ	G	G/ρ	H	V
Bond critical points						
Sb1-Se2	0.019	0.087	0.018	0.972	0.004	−0.014
Sb1-O3	0.195	0.775	0.245	1.260	−0.052	−0.297
Sb1-O4	0.189	759	0.239	1.267	−0.050	−0.289
Sb1-O17	0.216	0.767	0.258	1.197	−0.067	−0.325
Sb1…O28	0.045	0.276	0.060	1.346	0.009	−0.051
O4…O28	0.019	0.089	0.020	1.089	0.002	−0.018
Ring critical points						
Sb1-O3-C5-C8-Se2	0.013	0.053	0.013	1.020	0.000	−0.013
Sb1-O4-C14-C11-Se2	0.012	0.052	0.011	0.925	0.001	−0.010
Sb1-O4-O28	0.018	0.104	0.022	1.225	0.004	−0.019
Sb25-O4-O28	0.018	0.104	0.022	1.225	0.004	−0.019

As can be seen, the values for the intramolecular Sb…Se (Sb1-Se2) interaction are similar to the values in the monomer and can be characterized as closed-shell interactions. The intramolecular Sb-O bonds (Sb1-O3, Sb1-O4 and Sb1-O17) should be considered as polar shared bonds like in the monomer (see above). The electron densities in the intermolecular Sb…O interactions (Sb1…O28) are higher than in the Sb…Se (Sb1-Se2) interactions and can be characterized as closed-shell Van der Waals interactions [41,47].

**Figure 14:**
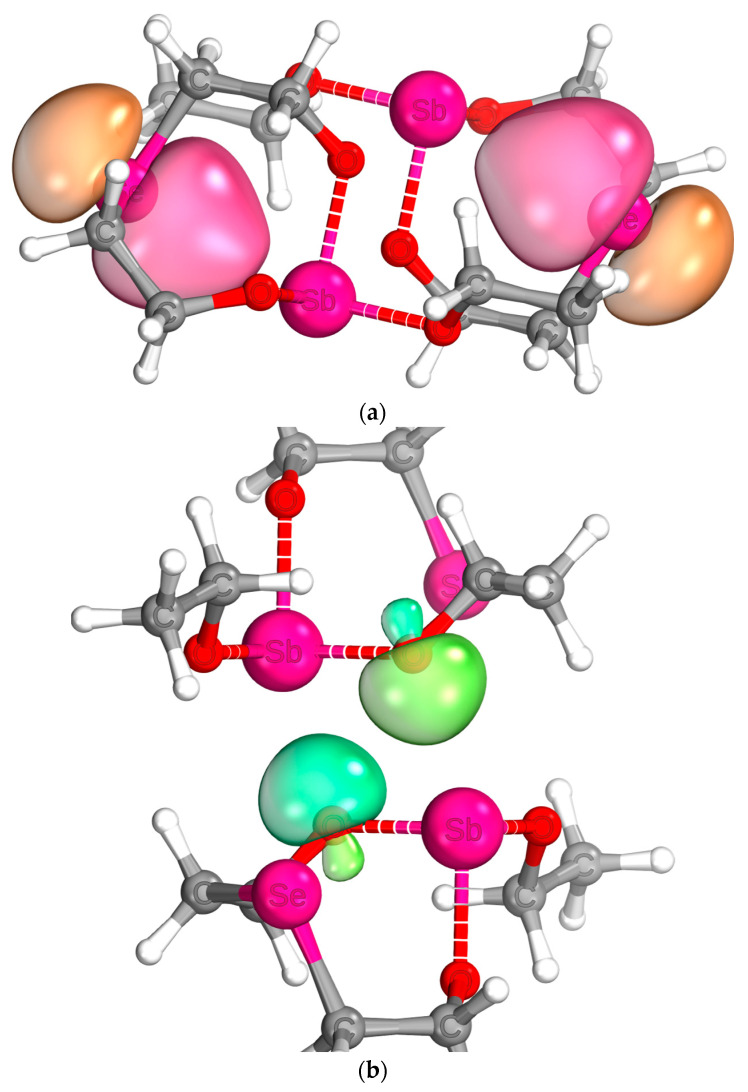

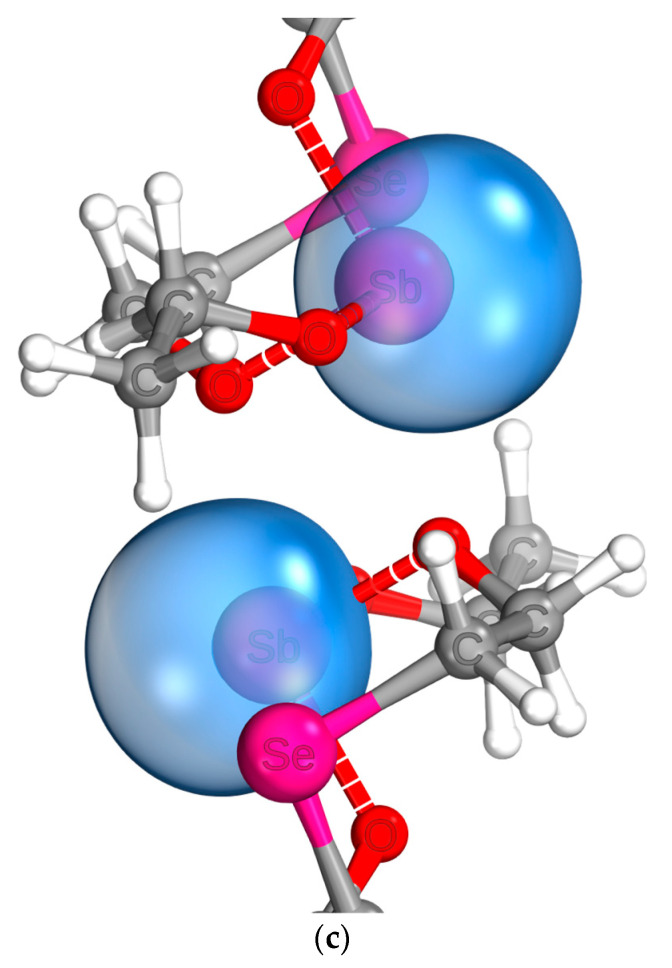
IBOs of a dimer of **3** for visualization of the intramolecular Sb…Se (**a**), and the intermolecular Sb…O (**b**) interactions, as well as the Sb lone pairs (**c**).

For the dimer, IBO [48] calculations were performed to visualize the Sb lone pairs as well as the Sb…O and Sb…Se interactions. Figure 14c shows, that the lone pairs at antimony are mostly spherical with an orientation to the outside of the Sb-O…Sb-O… plane forcing the geometry to be a parallelogram. There are orbitals mostly located at the O atoms, which are directed towards the Sb atom of the neighbouring molecule, which conforms the intermolecular Sb…O interactions (Figure 14b). Additionally, one of the orbitals located at each of the Se atoms are directed to the Sb atoms, confirming an intramolecular interaction of the atoms Se and Sb (Figure 14a).

### 2.6. Reactivity of **6**

Antimony occurs in two stable oxidation states in nature, +III and +V. In the presented complexes, the Sb atom should be in oxidation state +III. Therefore, there is a lone pair located at the Sb atom, and it can react as a Lewis base. Additionally, the electronegative atoms at the ligand causes σ-holes [50], and the complexes can react as Lewis acid, e.g., [51]. So, with the antimony(III) as Lewis acid and the donor atom E as a Lewis base within the same molecule, interesting reactivity could be expected. To investigate the reactivity exemplarily, compound **6** is used in further experiments, since it is soluble in most solvents like chloroform, acetone, ethanol, THF, and acetonitrile.

As nucleophiles, n-propylamine and ammonia gas were used. A nucleophilic attack to the antimony atom was expected. However, with these amines, no new species could be observed in the NMR spectra.

For redox reactions, elementary selenium and tellurium were added to the NMR sample of **6**. It could be expected that these chalcogens oxidize the antimony (III) to antimony (V). However, no reaction was observed after one week at room temperature.

**Scheme 1:**
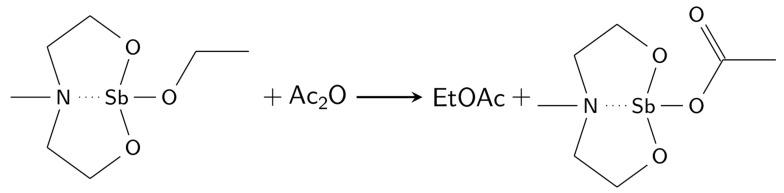
Reaction of **6** with acetic anhydride. Traces of acetic acid were present in the reaction mixture.

The reactions with electrophiles were studied with CO_2_ and acetic anhydride. These electrophiles can be attacked by the lone pair of the nitrogen atom or the lone pair of the antimony atom. With CO_2_, no reaction occurs. With acetic anhydride, new signals in the ^13^C NMR spectrum were found at 166.5, 170.7, 171.0, and a broad one at 178.3 ppm. These signals indicate the presence of acetic anhydride (166 ppm), ethyl acetate (171 ppm), and acetic acid (178 ppm). The signal at 170 ppm might indicate the formation of [MeN(CH_2_-CH_2_-O)_2_]Sb(OAc) with an acetyl instead of an ethoxy group. Sb(OAc)_3_ has a ^13^C NMR signal at 181 ppm and can therefore be excluded (see Supplementary Materials, Figures S1 and S2). Therefore, the reaction formulated in Scheme 1 can be assumed to occur in this experiment.

**Scheme 2:**
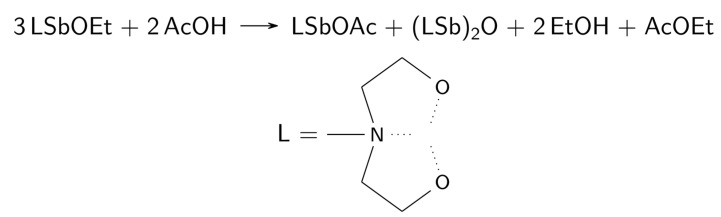
Reaction of 6 with acetic acid.

The reaction of **6** with acetic acid instead of acetic anhydride also leads to signals at 170 and 171 ppm in the ^13^C NMR spectrum, but with much lower intensity. It could be assumed that ethyl acetate is formed and a Sb-O-Sb dimer occurs as a joint product (see Scheme 2), but is not found in the NMR spectra because of the low intensity and very similar signals of the *N*-methyldiethanolamino ligand system.

The formation of ethyl formate is observed (signal at 161 ppm in the ^13^C NMR spectrum) in the reaction of **6** with formic acid. A white solid is formed in this reaction. This white solid is slightly soluble in chloroform, but more soluble in methanol. It is assumed that this solid is [MeN(CH_2_-CH_2_-O)_2_]Sb(O_2_CH) with a formyl moiety instead of an ethoxy moiety with a signal at 165 ppm in the ^13^C NMR spectrum (see Figure S15 in the Supplementary Materials).

## 3. Materials and Methods

### 3.1. General Considerations

All reactions were carried out under argon using the standard Schlenk technique [52,53]. Used chemicals and purification methods can be found in the Supplementary Materials. NMR spectra were recorded in CDCl_3_ or C_6_D_6_ with TMS as internal standard either on a BRUKER DPX 400 spectrometer at 400.13, 100.61 and 79.49 MHz for ^1^H, ^13^C and ^29^Si NMR spectra or on a BRUKER AVANCE III 500 MHz spectrometer at 500.13, 125.76 and 99.36 MHz for ^1^H, ^13^C and ^29^Si NMR spectra, respectively. Raman spectra were measured with Bruker FT-Raman spectrometer RFS 100/S. The device works with an air-cooled Nd:YAG-Laser with a wavelength of 1064 nm and a nitrogen-cooled detector. The samples were filled in glass tubes and sealed with PTFE-paste. The intensity is listed as follows: vs. (very strong) 80–100% of the highest signal, s (strong), m (medium), w (weak), vw (very weak) next 20%, respectively. Infrared spectra were obtained using a Thermo Fisher Nicolet (Waltham, MA, USA) 380 FT-IR-Spectrometer with ATR attachment. The intensities were listed as those of the Raman measurements. Boiling points of liquid samples were measured as described in [54]. Melting points were measured using a Polytherm A hot stage microscope from Wagner and Munz with an attached 52II thermometer from Fluke.

### 3.2. Syntheses and Characterization

#### 3.2.1. Synthesis of Triethoxyantimony

First, 60.83 g (267 mmol) of freshly distilled SbCl_3_ (Merck, Darmstadt, Germany) was dissolved in 48.37 g (1.05 mol) abs. ethanol (VWR, stored over molecular sieves 3 Å). To the ice cooled solution, 95.27 g (941 mmol) triethylamine (Chemsolut, distilled from Na/benzophenone) was added. Afterward, 40 mL hexane was added, and a white solid was formed. The suspension was filtrated, and all volatiles were removed in vacuum from the filtrate. The residue is distilled to yield 55.45 g (216 mmol, 81%) of a colourless liquid. B.p.: 68 °C/0.171 mbar; ^1^H NMR (CDCl_3_, 400 MHz) δ [ppm] = 1.27 (t, ^3^J_H,H_ = 7.0 Hz, 9 H, CH_3_), 4.05 (q, ^3^J_H,H_ = 7.0 Hz, 6 H, CH_2_); ^13^C NMR (CDCl_3_, 100 MHz) δ [ppm] = 19.8 (CH_3_), 58.7 (CH_2_); IR ν [cm^−1^] = 2963.2 (w), 2918.9 (w), 2862.0 (w), 1477.3 (vw), 1441.6 (vw), 1378.9 (w), 1358.7 (vw), 1154.2 (vw), 1091.6 (m), 1033.7 (vs), 887.1 (m), 630.6 (m), 603.6 (w); Raman ν [cm^−1^] = 2966.8 (m), 2922.4 (vs), 2866.5 (s), 2746.9 (vw), 2704.5 (vw), 2604.2 (vw), 1476.1 (vw), 1451.0 (vw), 1383.5 (vw), 1360.4 (vw), 1283.2 (vw), 1098.1 (vw), 1063.4 (vw), 891.7 (vw), 805.0 (vw), 596.7 (vw), 324.8 (vw), 114.6 (vw).

#### 3.2.2. Synthesis of 2-[(2-Hydroxyethyl)selanyl]ethan-1-ol/selenium-diglycol

First, 2.051 g (54.2 mmol) sodium borohydride and 2.306 g (57.6 mmol) sodium hydroxide were dissolved in about 30 mL deoxygenated water. 1.345 g (17.0 mmol) selenium was added. The suspension was stirred overnight while the selenium was dissolved to form a white suspension. Then, 3.56 g (44.2 mmol) 2-chloroethanol in 60 mL of THF was added, and the solution was stirred overnight. Two phases were formed. The organic phase was washed with saturated NaCl solution. The aqueous phase was extracted three times with 40 mL chloroform each time. The combined organic phases were dried over Na_2_SO_4_. All volatiles were removed in vacuum and a pale yellow liquid was yielded (1.05 g; 6.2 mmol; 37%).^1^H NMR (D_2_O, 400 MHz) δ [ppm] = 2.67 (t, ^3^J_H,H_ = 6.7 Hz, 4 H, Se-CH_2_), 3.69 (t, ^3^J_H,H_ = 6.7 Hz, 4 H, O-CH_2_), 4.69 (s, 2 H, OH); ^13^C NMR (D_2_O, 100 MHz) δ [ppm] = 25.6 (Se-CH_2_), 61.3 (O-CH_2_); ^77^Se NMR(D_2_O, 76 MHz) δ [ppm] = 83.2 ppm; IR ν [cm^−1^] = 3282.4 (w), 2967.1 (w), 2923.7 (w), 2868.7 (w), 1405.0 (w), 1272.9 (w), 1188.0 (vw), 1150.4 (vw), 1062.6 (m), 1039.5 (vs), 998.0 (s), 929.6 (w), 879.4 (w), 802.3 (vw), 632.6 (m), 615.2 (m), 600.7 (m); Raman ν [cm^−1^] = 2966.8 (w), 2928.2 (vs), 2876.1 (m), 2718.0 (vw), 1464.5 (vw), 1424.0 (vw), 1281.3 (vw), 1202.2 (vw), 1046.0 (vw), 1007.4 (vw), 934.2 (vw), 882.1 (vw), 671.9 (w), 565.8 (w), 457.8 (vw), 347.9 (vw), 268.8 (vw), 205.2 (vw), 124.2 (vw).

#### 3.2.3. Synthesis of 2-[(2-Hydroxyethyl)tellanyl]ethan-1-ol/tellurium-diglycol

First, 2.36 g (62.4 mmol) sodium borohydride and 2.62 g (65.5 mmol) sodium hydroxide were dissolved in about 30 mL deoxygenated water and 5 mL THF. Additionally, 2.275 g (17.8 mmol) of tellurium was added. The suspension is stirred at about 40 °C for three days while the tellurium is dissolved to form a white suspension. Then, 3.337 g (41.4 mmol) 2-chloroethanol in 10 mL of THF was added, and the solution was stirred overnight. Two phases were formed. The organic phase was washed with saturated NaCl solution. The aqueous phase was extracted three times with 40 mL chloroform each time. The combined organic phases were dried over Na_2_SO_4_. All volatiles were removed in vacuum and a yellow liquid was yielded (1.49 g; 6.8 mmol; 38%).

^1^H NMR (D_2_O, 400 MHz) δ [ppm] = 2.79 (t, ^3^J_H,H_ = 8 Hz, 4 H, Te-CH_2_), 3.81 (t, ^3^J_H,H_ = 8 Hz, 4 H, O-CH_2_), 4.68 (s, 2 H, OH); ^13^C NMR (D_2_O, 100 MHz) δ [ppm] = 6.0 (Te-CH_2_), 63.6 (O-CH_2_); ^125^Te NMR(Acetone-d_6_, 126) δ [ppm] = 127.7 (Product), 158.0 (ditelluride) ppm; IR ν [cm^−1^] = 2954.6 (w), 2930.4 (w), 2918.9 (w), 2875.5 (w), 2845.6 (m), 2822.5 (m), 2685.5 (w), 1471.5 (w), 1456.1 (w), 1444.5 (w), 1397.2 (m), 1375.1 (w), 1363.5 (w), 1273.8 (m), 1263.2 (m), 1192.8 (w), 1155.2 (w), 1091.6 (w), 1067.5 (s), 1054.9 (s), 1038.5 (vs), 986.5 (s), 960.4 (s), 918.0 (s), 897.7 (s), 804.2 (w), 786.9 (m), 756.0 (m), 666.3; (w); Raman ν [cm^−1^] = 2970.6 (w), 2928.2 (vs), 2874.2 (m), 2746.9 (vw), 2714.1 (vw), 1466.4 (vw), 1408.6 (vw), 1354.6 (vw), 1265.9 (vw), 1155.9 (w), 1088.4 (vw), 1040.2 (vw), 999.7 (vw), 922.6 (vw), 880.2 (vw), 625.6 (m), 513.8 (w), 434.7 (w), 324.8 (w), 191.7 (vw), 155.1 (vw), 124.2 (vw).

#### 3.2.4. Synthesis of 2-Ethoxy-1,3,6,2-trioxastibocane (**1**)

First, 2.13 g (20.1 mmol) diethyleneglycole was mixed with 5.13 g triethoxyantimony (20.0 mmol) in 20 mL cyclohexane. The mixture was heated until the head temperature rose to 80 °C (b.p. of cyclohexane). All volatiles were removed in vacuum and the white solid residue was recrystallized from chloroform. Yield: 4.99 g (18.4 mmol; 92%) of white crystals, suitable for single crystal X-ray diffraction.

^1^H NMR (CDCl_3_, 400 MHz) δ [ppm] = 1.27 (t, ^3^J_H,H_ = 8.0 Hz, 3 H, CH_3_), 3.35–4.43 (m, 10 H, all other H) ; ^13^C NMR (CDCl_3_, 100 MHz) δ [ppm] = 19.9 (CH_3_), 58.3 (O-CH_2_), 61.9 (CH_2_ of EtO), 71.6 (CH_2_-O-Sb); IR ν [cm^−1^] = 2955.5 (vw), 2901.5 (w), 2848.5 (w), 1466.7 (vw), 1440.6 (vw), 1373.1 (w), 1359.6 (w), 1272.9 (vw), 1250.7 (vw), 1241.0 (w), 1141.7 (vw), 1119.5 (w), 1072.3 (s), 1054.0 (s), 1035.3 (vs), 1009.6 (vs), 915.1 (m), 893.9 (m), 870.7 (vs), 811.9 (m); Raman ν [cm^−1^] = 2989.9 (vw), 2957.1 (m), 2926.3 (vs), 2914.7 (vs), 2870.4 (s), 2854.9 (s), 2739.2 (vw), 2704.5 (vw), 2679.4 (vw), 2584.9 (vw), 2565.7 (vw), 1456.8 (vw), 1449.1 (vw), 1379.6 (vw), 1360.4 (vw), 1343.0 (vw), 1275.5 (vw), 1254.3 (vw), 1142.4 (vw), 1123.2 (vw), 1092.3 (vw), 1074.9 (vw), 1059.5 (vw), 1049.9 (vw), 1011.3 (vw), 916.8 (vw), 899.4 (vw), 876.3 (vw), 814.6 (vw), 575.5 (vw), 546.5 (vw), 509.9 (w), 469.4 (w), 432.8 (vw), 344.0 (vw), 309.3 (vw), 278.5 (vw), 241.8 (vw), 199.4 (vw), 187.8 (vw), 158.9 (vw), 116.5 (vw); m.p. = 125 °C.

#### 3.2.5. Synthesis of 2-Ethoxy-1,3,6,2-dioxathiastibocane (**2**)

First, 2.33 g (19.1 mmol) 2.2′-thiodiglycol was mixed with 4.899 g triethoxyantimony (19.1 mmol) in 20 mL cyclohexane. The mixture was heated until the head temperature rose to 80 °C (b.p. of cyclohexane). All volatiles were removed in vacuum and the white slid residue was recrystallized from chloroform. Yield: 5.11 g (17.8 mmol; 93%) of white crystals, suitable for single crystal X-ray diffraction.

^1^H NMR (CDCl_3_, 500 MHz) δ [ppm] = 1.27 (t, ^3^J_H,H_ = 8.0 Hz, 3 H, CH_3_), 2.29–2.95 (m, 4 H, S-CH_3_), 3.97–4.51 (m, 6 H, O-CH_2_ (both)); ^13^C NMR (CDCl_3_, 125 MHz) δ [ppm] = 19.9 (CH_3_), 37.5 (O-CH_2_), 58.0 (CH_2_-O-Sb), 62.0 (CH_2_ of EtO); IR ν [cm^−1^] = 3319.1 (vw), 2953.6 (vw), 2912.1 (vw), 2844.6 (w), 2701.9 (vw), 1458.0 (w), 1445.5 (w), 1405.9 (w), 1399.2 (w), 1369.3 (w), 1362.5 (w), 1283.5 (w), 1224.6 (w), 1194.7 (w), 1178.4 (w), 1064.6 (s), 1033.7 (vs), 998.0 (vs), 940.2 (m), 890.0 (m), 837.0 (m), 807.1 (m), 729.0 (w), 694.3 (m), 649.9 (m), 612.3 (w); Raman ν [cm^−1^] = 2957.1 (w), 2914.7 (vs), 2853.0 (m), 2799.0 (vw), 2737.3 (vw), 2704.5 (vw), 1449.1 (vw), 1404.7 (vw), 1368.1 (vw), 1285.1 (vw), 1231.1 (vw), 1223.4 (vw), 1196.4 (vw), 1181.0 (vw), 1096.2 (vw), 1084.6 (vw), 1042.2 (vw), 999.7 (vw), 949.6 (vw), 895.6 (vw), 841.6 (vw), 810.7 (vw), 695.0 (vw), 652.6 (vw), 590.9 (vw), 550.4 (vw), 521.5 (vw), 509.9 (vw), 484.8 (vw), 459.8 (w), 384.5 (vw), 346.0 (vw), 313.2 (vw), 299.7 (vw), 255.3 (vw), 234.1 (vw), 214.8 (vw), 199.4 (vw), 164.7 (vw), 120.3 (vw); m.p. = 132 °C.

#### 3.2.6. Synthesis of 2-ethoxy-1,3,6,2-dioxaselenastibocane (3)

First, 1.285 g triethoxyantimony (5.0 mmol) was dissolved in 5 mL abs. ethanol. Next, 0.600 g selenium-diglycole (3.5 mmol) was added. After a few minutes, colourless crystals were obtained, suitable for single crystal X-ray diffraction. Yield: 0.963 g (2.9 mmol; 83%) of white crystals.

^1^H NMR (CDCl_3_, 500 MHz) δ [ppm] = 1.27 (t, ^3^J_H,H_ = 8.0 Hz, 3 H, CH_3_), 2.44–2.96 (m, 4 H, Se-CH_2_), 4.05 (q, ^3^J_H,H_ = 8.0 Hz, 2 H, CH_2_ of EtO) 4.11–4.64 (m, 4 H, O-CH_2_); ^13^C NMR (CDCl_3_, 125 MHz) δ [ppm] = 19.8 (CH_3_), 32.2 (O-CH_2_), 58.7 (CH_2_-O-Sb), 62.3 (CH_2_ of EtO); ^77^Se NMR (CDCl_3_, 76 MHz) δ [ppm] = 50.2; IR ν [cm^−1^] = 3278.6 (w), 2954.6 (vw), 2912.1 (w), 2841.7 (w), 2702.9 (w), 1699.1 (vw), 1455.1 (w), 1398.2 (m), 1361.6 (m), 1277.7 (m), 1211.1 (w), 1197.6 (w), 1179.3 (w), 1165.8 (w), 1062.6 (s), 1038.5 (vs), 1007.7 (vs), 976.8 (vs), 929.6 (s), 889.1 (m), 816.7 (m), 785.9 (m), 667.3 (m), 612.3 (w); Raman ν [cm^−1^] = 2980.3 (w), 2968.7 (w), 2922.4 (vs), 2893.5 (w), 2851.1 (m), 2791.3 (vw), 2706.4 (vw), 1449.1 (vw), 1402.8 (vw), 1370.0 (vw), 1289.0 (vw), 1277.4 (vw), 1213.8 (vw), 1196.4 (vw), 1179.1 (vw), 1082.7 (vw), 1049.9 (vw), 1015.2 (vw), 976.6 (vw), 936.1 (vw), 789.5 (vw), 671.9 (vw), 589.0 (vw), 575.5 (w), 500.3 (w), 469.4 (vw), 452.0 (w), 374.9 (vw), 320.9 (vw), 284.3 (vw), 257.3 (vw), 216.8 (vw), 199.4 (vw), 157.0 (vw), 116.5 (vw); m.p. = 109 °C.

#### 3.2.7. Synthesis of 2-Ethoxy-1,3,6,2-dioxatellurastibocane (**4**)

First, 1.54 g triethoxyantimony (6 mmol) was dissolved in 5 mL abs. ethanol. Next, 1.49 g tellurium-diglycole (6.8 mmol) was added. After a few minutes, colourless crystals were obtained, suitable for single crystal X-ray diffraction. The solid was filtrated and washed 3 times with 2 mL of ethanol. After removing volatiles in vacuum, 0.91 g (2.4 mmol; 40%) of a white powder was yielded. Crystals in the solution decompose in a few months to a black residue and a yellowish solution.

^1^H NMR (CDCl_3_, 400 MHz) δ [ppm] = 1.72 (t, ^3^J_H,H_ = 6.9 Hz, 3 H, CH_3_), 2.74 (m, 4 H, Te-CH_2_), 4.06 (q, ^3^J_H,H_ = 7.0 Hz, 2 H, CH_2_ of EtO), 4.19–4.82 (m, 4 H, O-CH_2_); ^13^C NMR (CDCl_3_, 100 MHz) δ [ppm] = 16.0 (CH_3_), 19.9 (O-CH_2_), 58.4 (CH_2_-O-Sb), 64.1 (CH_2_ of EtO); ^125^Te NMR (CDCl_3_, 126 MHz) δ [ppm] = 1.3 (br); IR ν [cm^−1^] = 2954.6 (w), 2930.4 (w), 2918.9 (w), 2875.5 (w), 2845.6 (m), 2822.5 (m), 2685.5 (w), 1471.5 (w), 1456.1 (w), 1444.5 (w), 1397.2 (m), 1375.1 (w), 1363.5 (w), 1273.8 (m), 1263.2 (m), 1192.8 (w), 1155.2 (w), 1091.6 (w), 1067.5 (s), 1054.9 (s), 1038.5 (vs), 986.5 (s), 960.4 (s), 918.0 (s), 897.7 (s), 804.2 (w), 786.9 (m), 756.0 (m), 666.3 (w); Raman ν [cm^−1^] = 2976.4 (m), 2920.5 (vs), 2853.0 (s), 2835.6 (s), 2702.6 (w), 1454.9 (vw), 1397.0 (vw), 1362.3 (vw), 1267.8 (vw), 1192.6 (vw), 1175.2 (vw), 1078.8 (vw), 631.4 (vw), 598.6 (vw), 535.0 (w), 494.5 (m), 469.4 (s), 444.3 (w), 423.1 (vw), 322.8 (w), 278.5 (vw), 189.8 (m), 143.5 (w); m.p. = 105 °C (decomp.).

#### 3.2.8. Synthesis of 2-Ethoxy-1,3,6,2-dioxazastibocane (**5**)

First, 2.20 g (20.9 mmol) diethanolamine was mixed with 4.988 g triethoxyantimony (19.4 mmol) in 20 mL cyclohexane. The mixture was heated until the head temperature rose to 80 °C (b.p. of cyclohexane). All volatiles were removed in vacuum and the white solid residue was recrystallized from chloroform. Yield: 4.82 g (17.9 mmol; 92%) of white powder, slightly contaminated with a residue of cyclohexane.

^1^H NMR (CDCl_3_, 400 MHz) δ [ppm] = 1.29–1.52 (m, 8 H, CH_3_ and cyclohexane), 1.89–2.73 (m, 4 H, N-CH_3_), 3.64–4.15 (m, 4 H, O-CH_2_), 4.36 (q, ^3^J_H,H_ = 8.0 Hz, 2 H, O-CH_2_ of EtO); ^13^C NMR (CDCl_3_, 100 MHz) δ [ppm] = 21.3 (CH_3_), 57.8 (N-CH_2_), 63.1 (CH_2_ of EtO), 78.1 (CH_2_-O); IR ν [cm^−1^] = 3203.3 (w), 2898.6 (w), 2839.8 (m), 2694.2 (w), 1446.4 (w), 1358.7 (w), 1125.3 (w), 1087.7 (m), 1043.4 (vs), 1013.5 (vs), 916.1 (m), 871.7 (s), 819.6 (w), 667.3 (m), 653.8 (m), 612.3 (m); Raman ν [cm^−1^] = 2916.6 (vs), 2858.8 (vs), 2733.4 (vw), 2696.8 (vw), 1452.9 (w), 1368.1 (vw), 1350.7 (vw), 1263.9 (vw), 1240.8 (vw), 1150.2 (vw), 1125.1 (vw), 1096.2 (vw), 1063.4 (vw), 1017.1 (vw), 920.7 (vw), 876.3 (vw), 820.4 (vw), 801.1 (vw), 531.1 (s), 450.1 (vw), 419.3 (vw), 388.4 (vw), 293.9 (vw), 205.2 (w), 114.6 (vw); m.p. could not be determined, because of waxy consistency.

#### 3.2.9. Synthesis of 2-Ethoxy-6-methyl-1,3,6,2-dioxazastibocane (**6**)

First, 2.668 g (22.4 mmol) *N*-methyldiethanlamine were mixed with 5.801 g triethoxyantimony (22.6 mmol) in 20 mL cyclohexane. The mixture was heated until the head temperature rose to 80 °C (b.p. of cyclohexane). All volatiles were removed in vacuum and the white slid residue was recrystallized from chloroform. Yield: 5.770 g (20.3 mmol; 90%) of white crystals, suitable for single crystal X-ray diffraction.

^1^H NMR (CDCl_3_, 400 MHz) δ [ppm] = 1.18–1.29 (m, 3 H, CH_3_), 2.54 (s, 3 H, N-CH_3_), 2.56–2.85 (m, 4 H, N-CH_2_), 3.94–4.32 (m, 6 H, O-CH_2_(both)); ^13^C NMR (CDCl_3_, 100 MHz) δ [ppm] = 20.2 (CH_3_), 41.7 (CH_3_-N), 57.5 (CH_2_ of EtO and N-CH_2_), 61.7 (CH_2_-O); IR ν [cm^−1^] = 2946.8 (vw), 2893.8 (vw), 2859.1 (vw), 2825.3 (vw), 2683.6 (vw), 1460.9 (vw), 1449.3 (w), 1416.5 (w), 1373.1 (w), 1361.6 (w), 1352.9 (vw), 1338.4 (vw), 1287.3 (vw), 1266.1 (w), 1259.4 (w), 1237.2 (vw), 1204.4 (vw), 1161.0 (vw), 1142.7 (vw), 1067.5 (s), 1042.4 (vs), 1030.8 (vs), 991.3 (s), 913.2 (w), 879.4 (vs), 870.7 (s), 757.0 (m), 675.0 (w); Raman ν [cm^−1^] = 2961.0 (s), 2951.4 (s), 2907.0 (vs), 2862.6 (vs), 2837.6 (vs), 2808.6 (w), 2733.4 (vw), 2712.2 (vw), 2694.9 (vw), 2669.8 (vw), 1470.3 (w), 1454.9 (vw), 1418.2 (vw), 1375.8 (vw), 1350.7 (vw), 1289.0 (vw), 1262.0 (vw), 1238.9 (vw), 1206.1 (vw), 1163.7 (vw), 1144.4 (vw), 1090.4 (vw), 1071.1 (vw), 1049.9 (vw), 1034.4 (vw), 995.9 (vw), 914.9 (vw), 885.9 (vw), 872.5 (vw), 758.7 (vw), 560.0 (vw), 521.5 (w), 500.3 (w), 440.5 (vw), 401.9 (vw), 361.4 (vw), 303.5 (vw), 272.7 (vw), 228.3 (vw), 189.8 (vw), 137.7 (vw); m.p. = 82 °C.

#### 3.2.10. Synthesis of 2-Ethoxy-1,4-oxathiastibpentan (**7**)

First, 1.583 g (6.2 mmol) triethoxyantimony was dissolved in 5 mL ethanol and 0.520 g (6.6 mmol) 2-mercaptoethanol was added. Within 2 h, white crystals were formed. The solid was filtrated and washed three times with fresh 2 mL ethanol. After removing all volatiles in vacuum, 0.85 g (3.5 mmol; 56%) white crystals, suitable for single crystal X-ray diffraction, were received.

^1^H NMR (CDCl_3_, 400 MHz) δ [ppm] = 1.27 (t, ^3^J_H,H_ = 6.96 Hz, 3 H, CH_3_), 3.10 (m, 2 H, S-CH_2_), 4.01 (q, ^3^J_H,H_ = 6.93 Hz, 2 H, CH_2_ of EtO); 4.39 (m, 2 H, O-CH_2_); ^13^C NMR (CDCl_3_, 100 MHz) δ [ppm] = 19.7 (CH_3_), 35.0 (S-CH_2_), 59.5 (CH_2_ of EtO), 71.5 (br, CH_2_-O); IR ν [cm^−1^] = 2910.4 (w), 2844.6 (m), 1456.1 (w), 1414.6 (w), 1378.0 (w), 1349.0 (w), 1269.0 (m), 1206.3 (w), 1170.6 (w), 1089.6 (w), 1033.7 (vs), 1000.0 (vs), 932.5 (m), 879.4 (s), 841.8 (s), 754.1 (w), 660.2 (s), 609.4 (m); Raman ν [cm^−1^] = 2949.4 (m), 2914.7 (vs), 2853.0 (m), 2824.1 (vw), 2746.9 (vw), 2704.5 (vw), 2685.2 (vw), 1456.8 (vw), 1449.1 (vw), 1422.1 (vw), 1352.6 (vw), 1271.6 (vw), 1211.9 (vw), 1171.4 (vw), 1094.2 (vw), 1046.0 (vw), 1009.4 (vw), 939.9 (vw), 849.3 (vw), 666.1 (vw), 569.7 (vw), 540.8 (vw), 471.3 (w), 459.8 (w), 419.3 (w), 349.8 (m), 303.5 (vw), 282.3 (m), 228.3 (vw), 195.6 (vw), 164.7 (vw), 131.9 (w), 108.8 (vw); m.p. = 93 °C.

### 3.3. Reactivity Studies

First, 0.73 g **6** was dissolved in 4.672 g CDCl_3_. To 0.5 mL of this solution were added in four different NMR tubes:

(1) 0.075 g n-Propylamine;
(2) 0.121 g formic acid and a white residue was formed;
(3) Tip of a spatula elementar tellurium;
(4) Tip of a spatula elementar selenium.

Next, 0.52 g **6** was dissolved in 1.467 g acetonitril-*d*_3_. Through 0.5 mL of this solution, we introduced into separate NMR tubes:

(5) CO_2_ (Linde, 5.3);
(6) NH_3_ (Nippon Gases, 4.0).

Then, 0.56 g **6** was dissolved in 1.756 g CDCl_3_. To 0.5 mL of this solution, we added in separate NMR tubes:

(7) 0.2 mL acetic acid (freshly distilled from Ac_2_O);
(8) 0.2 mL acetic anhydride (freshly distilled).

### 3.4. Crystal Structure Analyses

Single crystal X-ray diffraction data of **1**–**4**, **6**, and **7** were collected on a STOE IPDS-II image plate diffractometer equipped with a low-temperature device with Mo-Kα radiation (λ = 0.71073 Å) using ω and φ scans. Crystal data and details of structure refinement are summarized in Table 6 and Table 7. Software for data collection was X-AREA, for cell refinement, X-AREA, and for data reduction, X-RED [55]. Preliminary structure models were derived by direct methods [56] and the structures were refined by full-matrix least-squares calculations based on F^2^ for all reflections using SHELXL [57]. All hydrogen atoms were included in the models in calculated positions and were refined as constrained to the bonded atoms. The ethoxy groups in the crystal structures of **2** and **3** are disordered and were refined with split atom models for the atoms C5 and C6. Parts A and B of the disordered ethyl groups have site occupation factors of 0.72/0.28 in compound **2** and 0.78/0.22 in compound **3**.

The crystal under investigation of compound **1** was twinned with three components (second component with 40.9%, third component with 1.8%). Overlap from the second and third domain causes errors in the intensities of some reflections, leading to spurious peaks and holes of residual electron density.

Compound **6** crystallizes with one molecule chloroform in the asymmetric unit.

The compounds form intermolecular interactions in the crystal lattice via Sb1…O1 and Sb1…O2 interactions. This leads to four membered rings of Sb1…O…Sb1A…O and causes short distances between neighbouring oxygen atoms. This leads to alert level A or B warnings in the CheckCIF routine [58]. These short contacts, O1…O1 and O2…O2, should not be considered as an error, but they are an intrinsic property of these solid state structures.

CCDC 2263270-2263275 contains the supplementary crystallographic data for this paper. These data can be obtained free of charge from The Cambridge Crystallographic Data Centre via www.ccdc.cam.ac.uk/data_request/cif.

### 3.5. Quantum Chemical Calculations

Initial geometries were optimized using the B97-3c [43] method as implemented in the ORCA software package version 5 [59]. The calculation of Hessian matrices verified the presence of local minima with zero imaginary frequencies. The vibration frequencies (infrared and Raman spectra) were calculated with the same method and scaled according to [60]. The B97-3c method is recommended because of the good cost–benefit-ratio for structure optimization and frequency calculations [61]. Additionally, a benchmark of the accuracy of the IR calculations performed with this method was made by Katysuba et al. showing that this method offers similarly good results as the well-recognized B3LYP functional [60]. The data of the experimental molecular structures (see Section 2.4) were used as a starting point for the geometry optimization. As no experimental molecular structure of 5 was obtained, the data of 6 were adopted and used. The RMSD of the optimized and experimental structures on all non-hydrogen atoms was calculated using the curcuma software package [62] and are below 0.4 Å (see Supplementary Materials).

Since quantum chemical calculations cannot output band widths and band forms for vibrations, a 10 cm^−1^ line width with a Gaussian line shape is assumed for comparison. Considering this, the fingerprints of experimental and calculated spectra look quite similar.

The atom coordinates of the optimized molecules can be found in the Supplementary Materials. The wave function for QTAIM calculations were performed at the PBE0 [44,45]/def2-TZVPP [46] level of theory with the NOECP option to exclude any pseudopotentials. The topological analysis was performed with the program Multiwfn [63]. IBO calculations were performed at PBE0/def2-TZVPP level of theory with the program IBOview [48].

## 4. Conclusions

A simple and straightforward method for the synthesis of antimony(III) complexes with tridentate *O*,*E*,*O*-ligands was developed. The complexes were synthesized from triethoxyantimony with the ligand in ethanol in good yields. Nitrogen and chalcogen atoms were used as donor atom E in the ligand system. In the Raman spectra, a ν(Sb-E) can be found as predicted with quantum mechanical methods.

The single crystal structure determination of compounds **1**–**4**, and **6** confirms the formation of chelate complexes, wherein the ligand molecules coordinate as tridentate *O*,*E*,*O*-ligands to the antimony atom. Furthermore, the formation of dimers in the solid state was observed in all these crystal structures. This allows the antimony atoms to reach pentacoordination. Compound **7** bears a bidentate ligand leading to the molecular composition (O-CH_2_-CH_2_-S)Sb(OEt). This triple-coordinated complex compensates for the coordinative unsaturation in the crystalline state by associating with two neighbouring molecules each and thus also achieves the fivefold coordination of antimony.

According to QTAIM calculations, there are polar shared bonds between antimony and the oxygen atoms of the ethoxy groups and between antimony and the lateral oxygen atoms of the chelate ligands. The interactions between the donor atom E and the Sb atom can be characterized as Van der Waals interactions. This could be confirmed by IBO analysis, which shows a displacement of orbitals located at the donor atom E towards the Sb atom. The formation of dimers in the solid state is observed and analysed. There are Sb…O Van der Waals interactions according to QTAIM and IBO analysis. Additionally, the IBO analysis shows that the lone pair of the antimony is located spherically around the Sb atom, which indicates a high s-character of this orbital.

Despite the lone pair at the antimony atom and the Lewis acid properties caused by the σ hole formation at the same atom [64], no reactions with nucleophiles were observed at room temperature. An oxidation of antimony(III) with selenium or tellurium was also not observed. The complexes react with acetic anhydride and more slowly with carboxylic acids, whereby the ethoxy moiety is substituted.

Further investigations on these complexes are part of the research in our group.

## Figures and Tables

**Figure 13:**
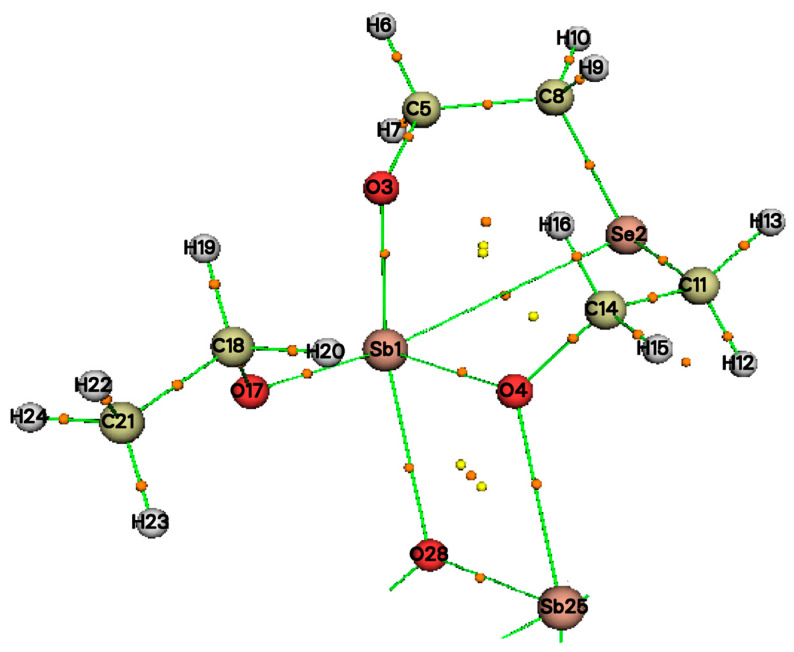
Atomic numbering scheme from quantum chemical calculation of the dimer of **3**. Only one molecule and O28 and Sb25 of the second molecule are shown (Remark: There is a BCP between H16 and O3 and a corresponding RCP in the Sb1-O4-C14-H16-O3 ring, which are quite near to the RCP of Sb1-O3-C5-C8-Se2 ring in this figure).

**Table 1:** Comparison between calculated (in brackets) and experimental Raman spectra. The index “cy” indicates inner-cyclic moieties. Frequencies are scaled as reported in [29].

Compound	ν(Sb-OEt)	δ(O-Sb-O)_cy_	ν_asym_(O-Sb-O)_cy_	ν_sym_(O-Sb-O)_cy_	ν(Sb…E)	ν_asym_(C-O)_cy_	ν_sym_(C-O)_cy_
**1**	576 (617)	188 (209)	510 (500)	546 (525)	159 (180)	1059 (1074)	1075 (1079)
**2**	591 (609)	199 (198)	485 (496)	521 (504)	165 (171)	1085 * (1074)	1085 * (1077)
**3**	589 (608)	199 (194)	469 (493)	500 (512)	117 (143)	1050 (1063)	1083 (1076)
**4**	599 (605)	190 (202)	469 (488)	495 (512)	143 (129)	1079 (1058)	1175 (1072)
**5**	531 * (605)	115 (191)	531 * (500)	531 * (518)	205 (217)	1063 (1064)	1096 (1088)
**6**	560 (604)	190 (201)	500 (501)	522 (517)	138 (163)	1050 (1071)	1071 (1084)
**7**	541 (614)	303 (316) ^1^	570 (559) ^2^	350 (365) ^3^			

^1^ δ(S-Sb-O)_cy_; ^2^ ν(Sb-O)_cy_; ^3^ ν(Sb-S)_cy_; * overlapping with other bands.

**Table 2:** Distances Sb…E for compounds **1**–**4**, and **6** from the molecule structures, sum of atomic radii and sum of Van der Waals radii (all values in Å).

Compound	E	Distance Sb…E	Sum of Atomic Radii ^a^	Sum of Van der Waals Radii ^b^
**6**	N	2.523(2)	2.11	3.61
**1**	O	2.776(7)	2.07	3.58
**2**	S	3.182(1)	2.45	3.86
**3**	Se	3.2856(6)	2.58	3.96
**4**	Te	3.5955(4)	2.78	4.12

^a^ Values for atomic radii taken from [39]: r(Sb) = 1.41, r(O) = 0.66, r(S) = 1.04, r(Se) = 1.17, r(Te) = 1.37 Å; ^b^ consistent values for Van der Waals-radii taken from [40]: r(Sb) = 2.06, r(O) = 1.52, r(S) = 1.80, r(Se) = 1.90, r(Te) = 2.06 Å.

**Table 3:** Angles C2-E-C3 (values in °).

Compound	E	Value
**6**	N	113.50 (16)
**1**	O	113.8 (8)
**2**	S	102.4 (2)
**3**	Se	99.43 (15)
**4**	Te	99.59 (16)

**Table 4:** Results of the topological analysis of **3** (all values in atomic units). Parameters are: charge density (ρ), its Laplacian (∇^2^ρ), kinetic energy density (G), total energy density (H), and electronic potential energy density (V). The atom numbering is the same as in Figure 8, resulting from the quantum chemical calculation file (see Supplementary Materials).

	ρ	∇^2^ρ	G	G/ρ	H	V
Bond critical points						
Sb1-Se2	0.019	0.090	0.019	0.977	0.004	−0.014
Sb2-O3	0.204	0.817	0.260	1.279	−0.056	−0.317
Sb1-O4	0.207	0.801	0.260	1.254	−0.060	−0.320
Sb1-O17	0.222	0.772	0.263	1.183	−0.070	−0.333
Ring critical points						
Sb1-O3-C5-C8-Se2	0.013	0.055	0.013	1.024	0.001	−0.012
Sb1-O4-C14-C11-Se2	0.012	0.054	012	0.947	0.002	−0.010

**Table 6:** Crystallographic data from data collection and refinement processes for **1**–**3**.

Compound	1	2	3
Formula	C_6_H_13_O_4_Sb	C_6_H_13_O_3_SSb	C_6_H_13_O_3_SbSe
M_r_	270.91	286.97	333.87
T (K)	173	173 K	193 K
λ (Å)	0.71073	0.71073 A	0.71073 A
Crystal system	Triclinic	Triclinic	Triclinic
Space group	P-1	P-1	P-1
a (Å)	6.8212(14)	6.8546(9)	6.8941(9)
b (Å)	8.1159(17)	8.9585(11)	9.0062(11)
c (Å)	9.1607(18)	8.9782(11)	9.1442(11)
α (°)	109.984(15)	111.722(9)	112.040(9)
β (°)	106.005(16)	103.710(10)	103.694(10)
γ (°)	100.526(16)	101.051(10)	100.022(10)
V (Å^3^)	436.07(16)	473.14(11)	489.04(11)
Z	2	2	2
ρ_calc_ (g·cm^−3^)	2.063	2.014	2.267
μ (mm^−1^)	3.131	3.097	6.504
F(000)	264	280	316
θ_max_ (°)	27.728	27.362	27.494
Reflections collected/unique [R_int_]	5772/5772	7414/2110 [R(int) = 0.0355]	7114/2125 [R(int) = 0.0171]
Completeness to θ = 25.242°	100.0%	99.9%	93.7%
Absorption correction	Integration	Integration	Integration
Max. and min. transmission	0.6891 and 0.3910	0.8413 and 0.4636	0.6084 and 0.3435
Data/restraints/parameters	5772/0/104	2110/3/121	2125/9/122
GoF on F^2^	1.108	1.135	1.179
Final R indices [I>2sigma(I)]	R_1_ = 0.0428, wR_2_ = 0.1036	R_1_ = 0.0253, wR_2_ = 0.0625	R_1_ = 0.0208, wR_2_ = 0.0523
R indices (all data)	R_1_ = 0.0473, wR_2_ = 0.1115	R_1_ = 0.0292, wR_2_ = 0.0648	R_1_ = 0.0220, wR_2_ = 0.0537
Extinction coefficient	0.012(2)	n/a	0.033(4)
Largest peak and hole (e·Å^−3^)	2.018 and −1.429	0.465 and −1.130	0.416 and −0.703

**Table 7:** Crystallographic data from data collection and refinement processes for **4**, **6**, and **7**.

Compound	4	6·HCCl_3_	7
Formula	C_12_H_26_O_6_Sb_2_Te_2_	C_7_H_16_NO_3_Sb·HCCl_3_	C_4_H_9_O_2_SSb
M_r_	765.03	403.32	242.92
T (K)	173	183	153
λ (Å)	0.71073	0.71073	0.71073
Crystal system	Monoclinic	Triclinic	Monoclinic
Space group	I2/a	P-1	P2(1)/n
a (Å)	12.8769(8)	7.1133(6)	6.9188(6)
b (Å)	12.8026(8)	9.8959(8)	6.7334(4)
c (Å)	13.7344(10)	11.0079(9)	15.7931(17)
α (°)	90	107.308(6)	90
β (°)	115.085(5)	99.987(6)	95.307(8)
γ (°)	90	94.568(6)	90
V (Å^3^)	2050.7(2)	721.44(11)	732.60(11)
Z	4	2	4
ρ_calc_ (g·cm^−3^)	2.478	1.857	2.202
μ (mm^−1^)	5.444	2.460	3.969
F(000)	1408	396	464
θ_max_ (°)	27.494	27.497 deg.	27.406 deg.
Reflections collected/unique [R_int_]	16987/2352 [R(int) = 0.0208]	12482/3264 [R(int) = 0.0228]	6249/1616 [R(int) = 0.0184]
Completeness to θ = 25.242°	99.5%	99.5%	97.1%
Absorption correction	Integration	Integration	Integration
Max. and min. transmission	0.5982 and 0.4041	0.8195 and 0.5737	0.6661 and 0.3293
Data/restraints/parameters	2352/0/101	3264/0/148	1616/0/75
GoF on F^2^	1.246	1.125	1.230
Final R indices [I>2sigma(I)]	R_1_ = 0.0232, wR_2_ = 0.0523	R_1_ = 0.0189, wR_2_ = 0.0485	R_1_ = 0.0236, wR_2_ = 0.0547
R indices (all data)	R_1_ = 0.0265, wR_2_ = 0.0545	R_1_ = 0.0202, wR_2_ = 0.0492	R_1_ = 0.0295, wR_2_ = 0.0604
Extinction coefficient	-	0.0238(12)	0.0108(10)
Largest peak and hole (e·Å^−3^)	1.077 and −0.885	0.698 and −0.540	0.575 and −0.621

## Data Availability

Data are contained within the article or Supplementary Materials.

## Supplementary Materials

The following Supplementary Materials can be downloaded at: https://www.mdpi.com/article/10.3390/molecules28134959/s1, Statement of origin and purification method of used chemicals; Table S1. NMR spectra (^1^H and ^13^C{^1^H}) of CDCl_3_ solutions of Sb(OAc)_3_ and 2; Figures S1–S4. IR and Raman spectra of 1 and 6; Figures S5 and S6. Additional figures of the molecular structures; Figures S7–S14. Further data of the topological analysis of compounds 1, 2, 4, 5, 6; Section S7. The atomic coordinates of all quantum chemically optimized structures; Section S8. The RMSD of the experimental and optimized structures; Section S9. The ^13^C NMR spectrum of the reaction product of **6** with formic acid; Figure S15.
