# Supplementary material for: New Complexes of Antimony(III) with Tridentate O,E,O-Ligands (E = O, S, Se, Te, NH, NMe) Derived from N-Methyldiethanolamine
Source: Molecules. 2023 Jun 24;28(13):4959. doi: 10.3390/molecules28134959 (PMC10343548; doi:10.3390/molecules28134959)
Supplement: Supplementary file 1 [file molecules-28-04959-s001.zip › molecules-2439349-supplementary.pdf]

Supplementary Materials for

New Complexes of Antimony(III) with Tridentate *O*,*E*,*O*-Ligands (E = O, S, Se, Te, NH, NMe) Derived from *N*-Methyldiethanolamine

Uwe Böhme and Marcus Herbig*

Institut für Anorganische Chemie, TU Bergakademie Freiberg, Leipziger Str. 29, 09599 Freiberg, Germany.

Content:

1. Origin and purification of chemicals

2. NMR Spectra of $Sb(OAc)_3$

3. NMR Spectra of **2**

4. IR and Raman Spectra of 1

5. Comparision of IR Spectra of N-ethyldiethanolamine and **6**

6. Crystal Structures

7. Additional data from AIM analysis

8. Coordinates of optimized molecules

9. RMSD of experimental and optimized structures on non-hydrogen atoms

10. ^{13}C NMR spectrum of the reaction product of **6** with formic acid

1. Origin and Purification of Chemicals

The chemicals used, their origin and the method applied for drying are listed in Table S1.

Table S1: Sources and purities of chemicals.

Chemical	Source, Purity	Purification
Ethanol	Fisher Scientific GmbH, 99,99 %	Column filled with molecular sieves 3 Å
Antimony(III)-chloride	Merck, p. A.	distillation
Chloroform-D	Deutero GmbH, 99,8 %	distillation from CaH_2
Acetonitril-D3	Deutero GmbH, 99,8 %	none
Triethylamine	ChemSolute, p. A.	distillation from Na/Benzophenone
Hexane	VWR, > 99 %	MBRAUN SPS 800
Sodium borohydride	Riedel-de-Häen, PROSYNTH	none
Sodium hydroxide	VWR International GmbH, p.a.	none
Selenium	Riedel-de-Häen, p. A.	none
2-Chloroethanol	Merck, p. A.	none
THF	VWR, >99 %	MBRAUN SPS 800
Chlorform (stabilized with amylene)	Fisher Scientific, 99 %	Column filled with molecular sieves 3 Å and activated alumina
Tellurium	Sigma-Aldrich, 99,8 %	none
Diethyleneglycol	Merck, 99 %	none
Cyclohexane	Fisher Scientific	Distillation from Na/Benzophenone
2,2-Thiodiglycol	Merck, ≥99%	none
Diethanolamine	Alfa-Aesar, 99 %	distillation

Chemical	Source, Purity	Purification
N-Methyldiethanolamine	Merck, ≥99 %,	distillation
2-Mercaptoethanol	ThermoFisher, 98 %	none
n-Propylamine	Merck, ≥99%	Distillation from CaH_2
Formic acid	ChemSolute, 99 %	none
Acetic acid	ChemSolute, 99,5 %	Distillation from Acetic anhydride
Acetic anhydride	ChemSolute, 99,5 %	distillation
CO_2 (Gas)	Linde, 5.3	Passing through a column filled with activated molecular sieves 3 Å
NH_3 (Gas)	Nippon Gases, 4.0	none
Ar (Gas)	Nippon Gases, 4.0	none

2. NMR spectra of $Sb(OAc)_3$

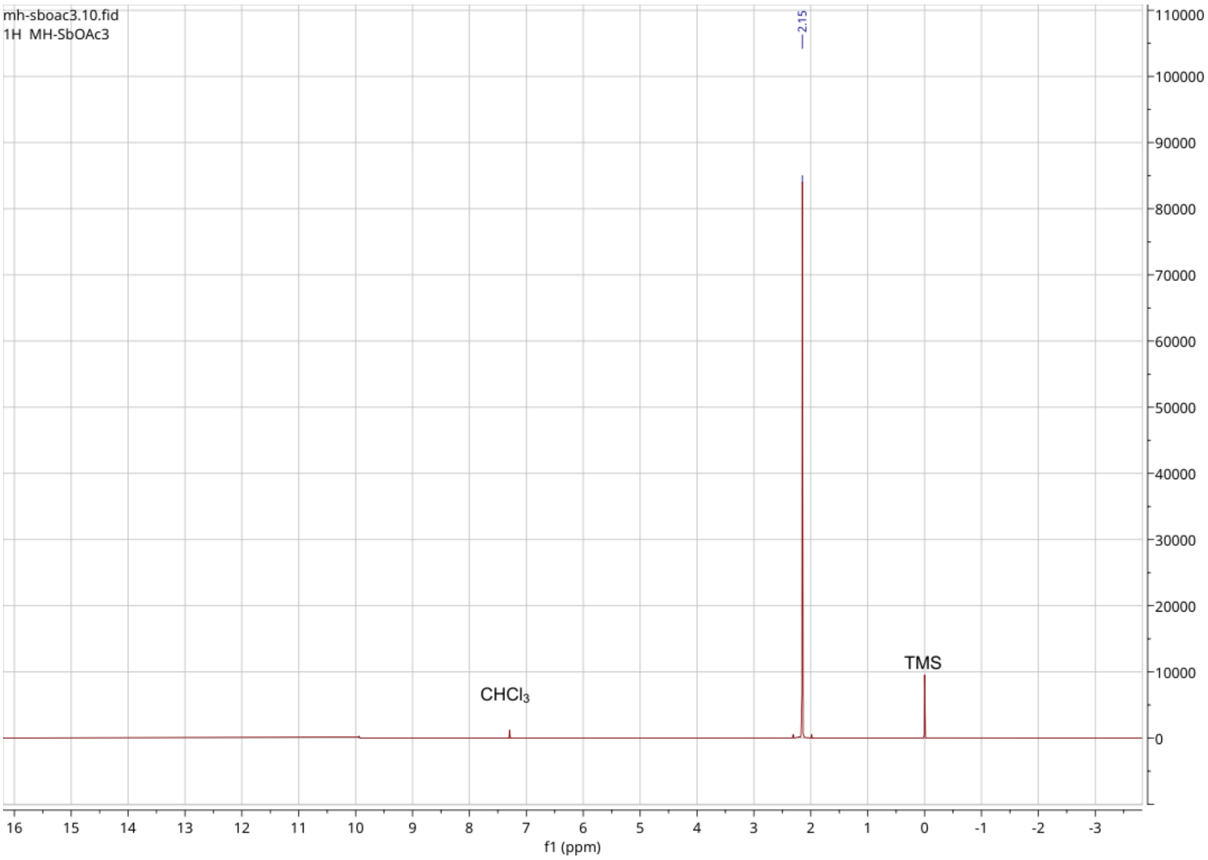

Figure S1: ^{1}H NMR spectrum of $Sb(OAc)_3$.

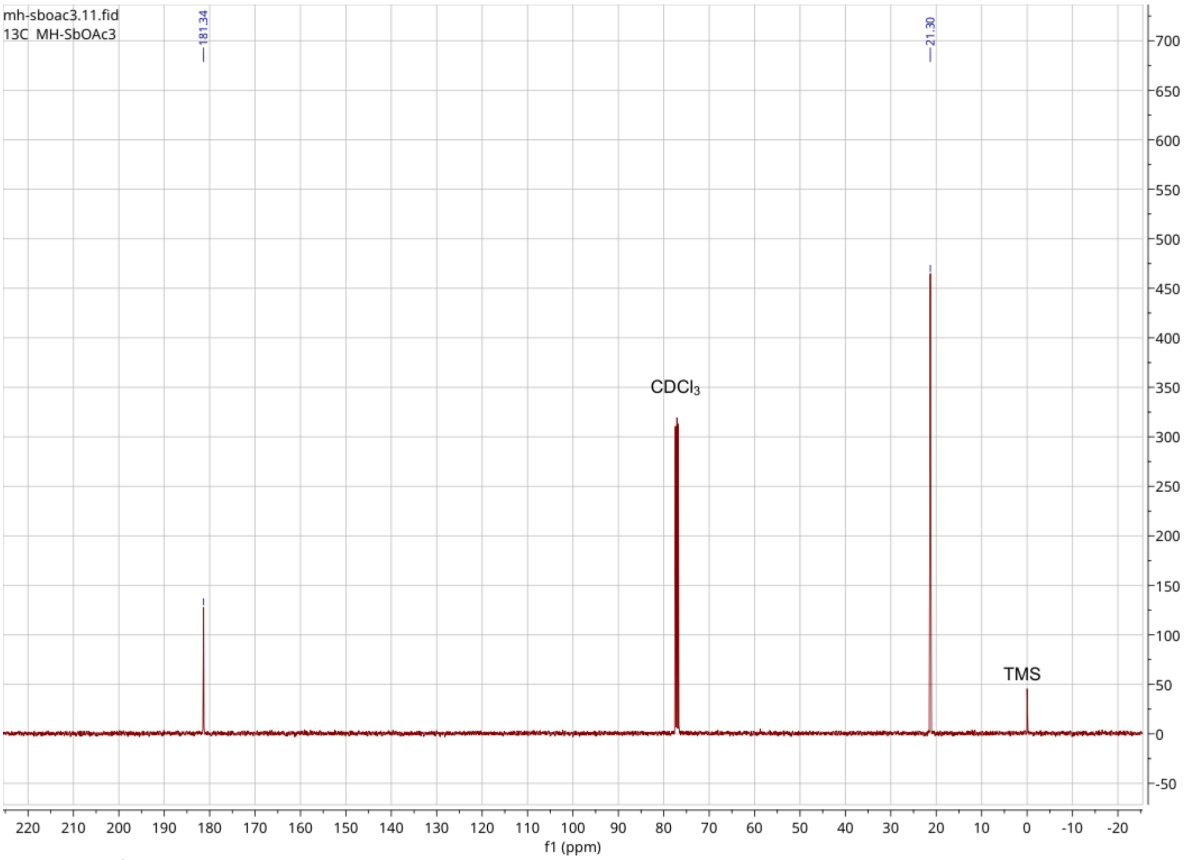

Figure S2: ^{13}C NMR spectrum of $Sb(OAc)_3$.

3. NMR Spectra of 2

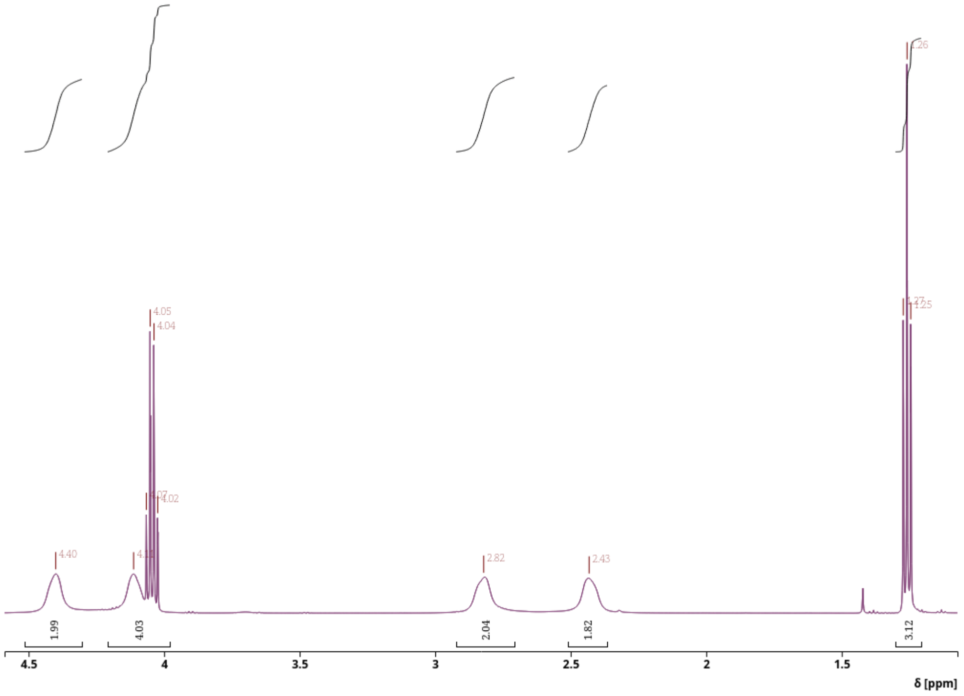

Figure S3: ^{1}H NMR spectrum of **2**. The signals of the protons of the tridentate ligand are split into two broad signals each, since the equatorial and axial positions are not equivalent any more in the ring system.

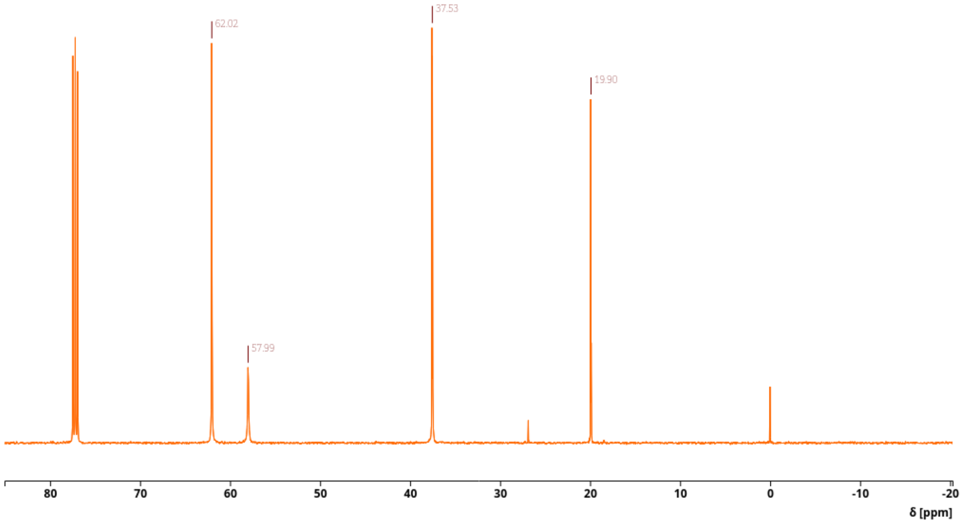

Figure S4: ^{13}C NMR spectrum of **2** (decoupled). The signal of the inner-cyclic O-CH_2 moiety becomes less intense and broader, most probably due to the proximity to the quadrupole Sb atom.

4. IR and Raman Spectra of 1

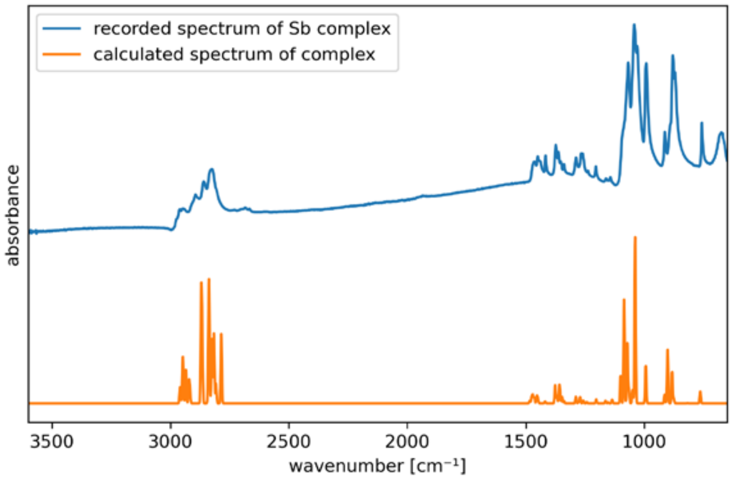

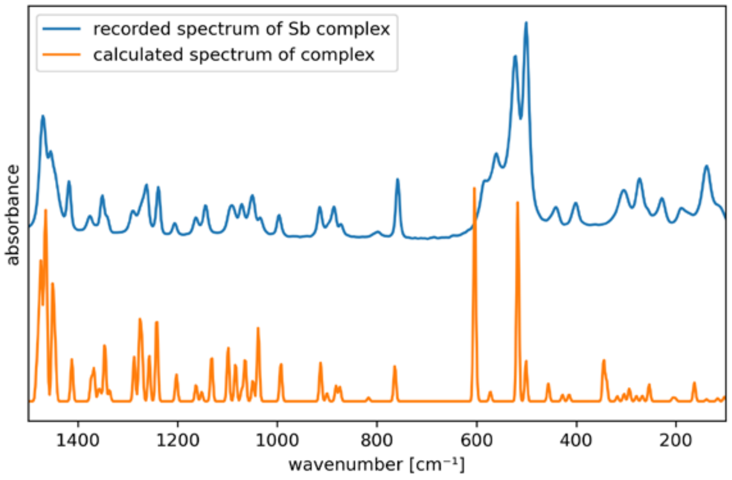

Figure S5: Comparison of recorded (blue) and calculated (orange) IR (top) and Raman (bottom) spectra for compound **6**. The calculated spectra were drawn with 10 cm^{-1} line width and Gaussian line shape and the data were scaled as reported in the literature (Katsyuba, S.A.; Zvereva, E.E.; Grimme, S. Fast Quantum Chemical Simulations of Infrared Spectra of Organic Compounds with the B97-3c Composite Method. J. Phys. Chem. A **2019**, 123 (17), 3802–3808, doi: 10.1021/acs.jpca.9b01688). For the Raman spectra, only the fingerprint range from 50 to 1500 cm^{-1} is shown since the vibrations having higher wave numbers are of a higher intensity.

5. Comparision of IR Spectra of *N*-ethyldiethanolamine and 6

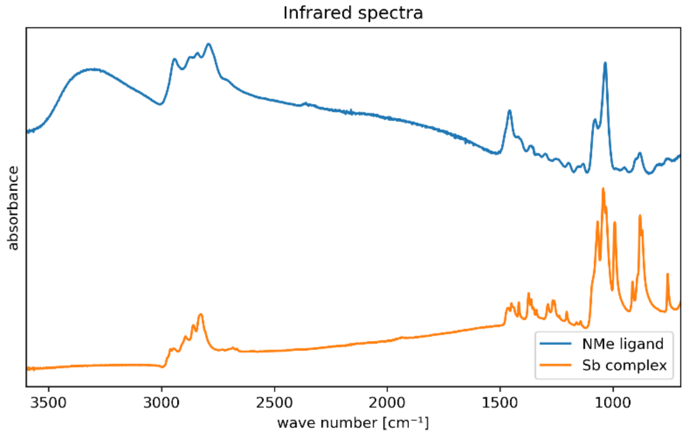

Figure S6: Infrared spectra of the ligand *N*-methyldiethanolamine (blue) and the corresponding antimony complex **6** (orange) in the range from 600 to 3600 cm^{-1}.

6. Crystal Structures

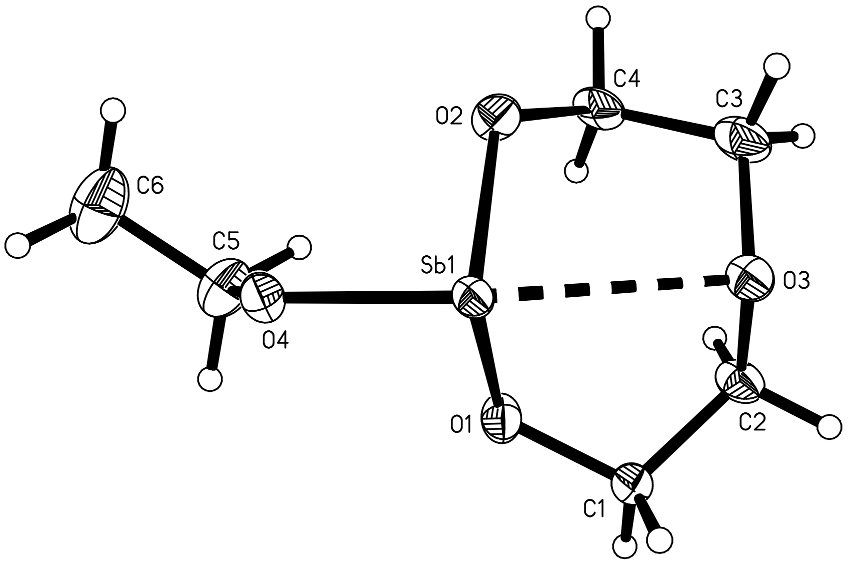

Figure S7: Asymmetric unit of **1** with atomic numbering scheme, shown with 50 % probability ellipsoids.

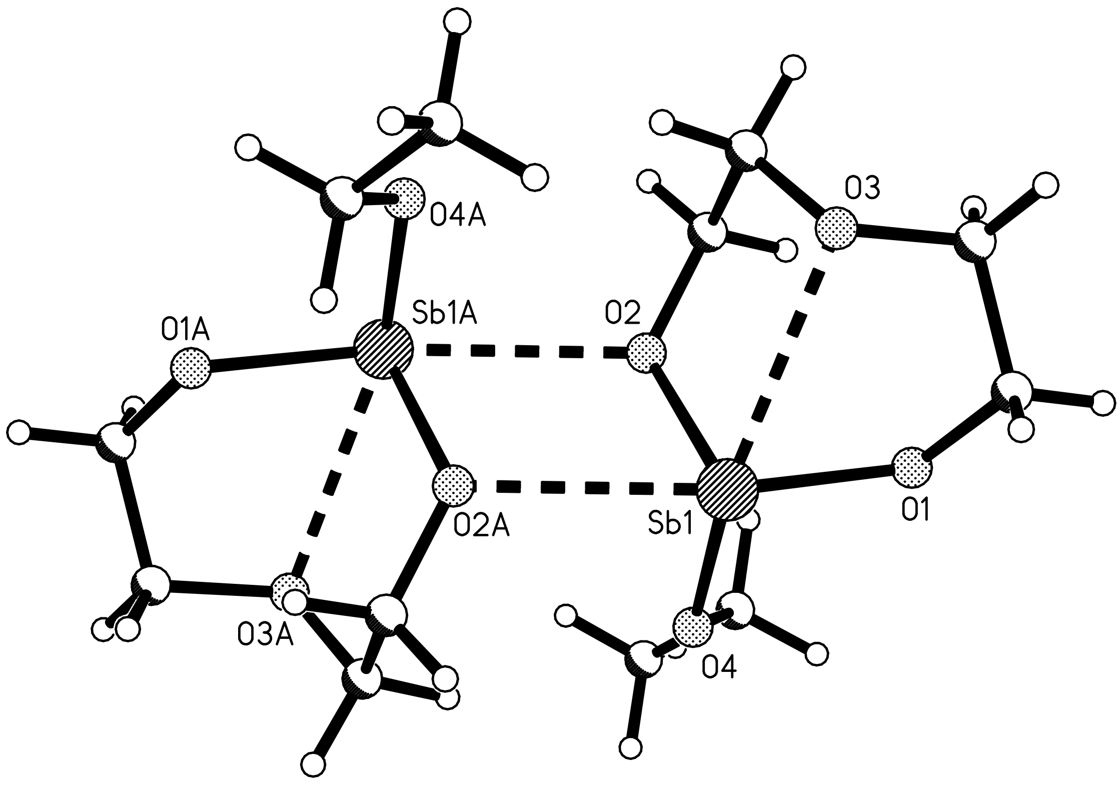

Figure S8: Dimer of **1** in the crystal structure. (The symmetry equivalent molecule is generated by symmetry operation 2-x, 1-y, 1-z.)

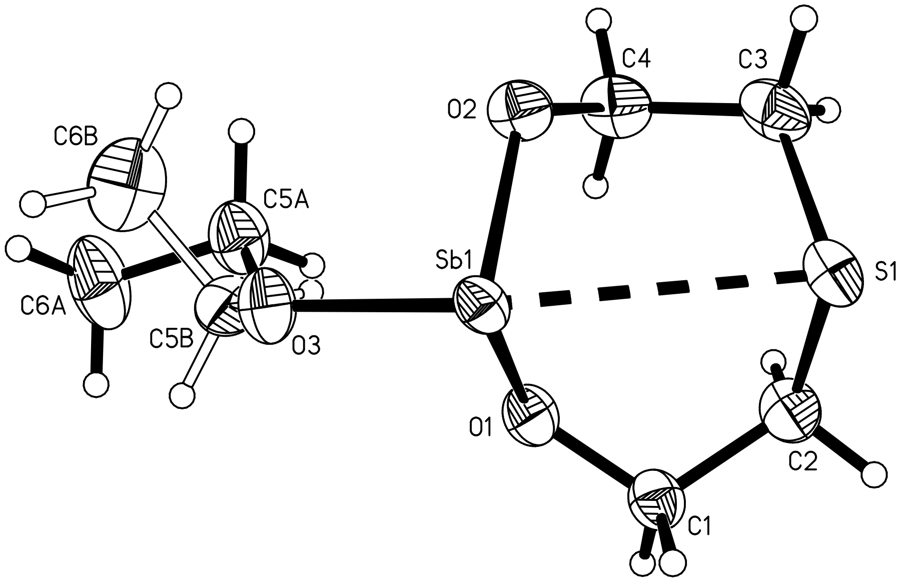

Figure S9: Asymmetric unit of **2** with atomic numbering scheme, shown with 50 % probability ellipsoids.

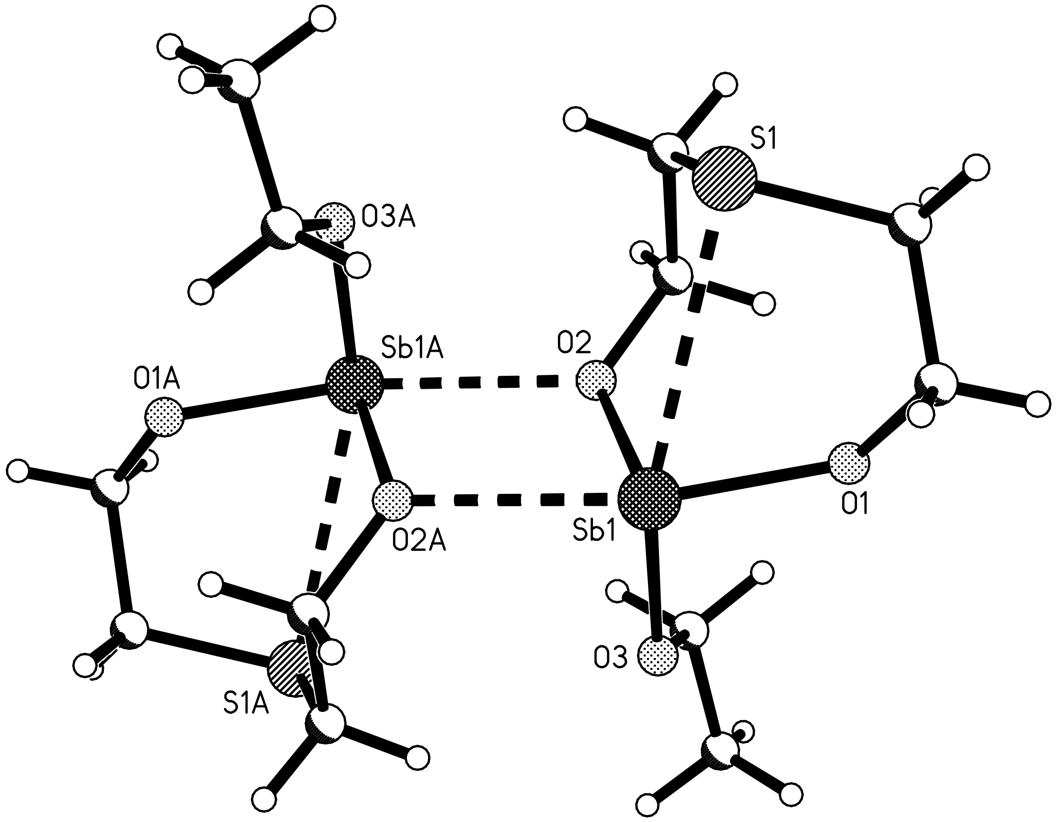

Figure S10: Dimer of **2** in the crystal structure. (The symmetry equivalent molecule is generated by symmetry operation 1-x, 1-y, 1-z.)

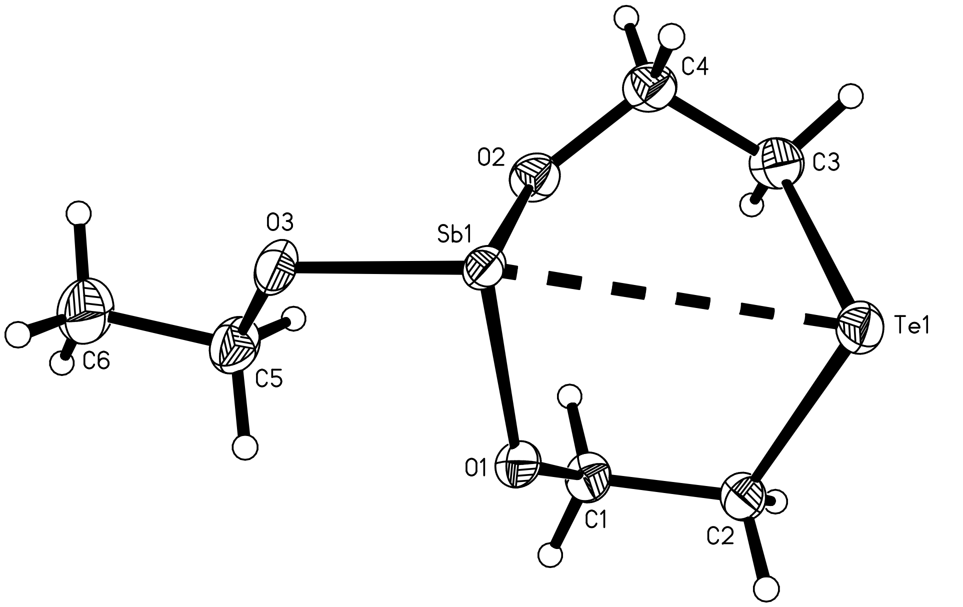

Figure S11: Asymmetric unit of **4** with atomic numbering scheme, shown with 50 % probability ellipsoids.

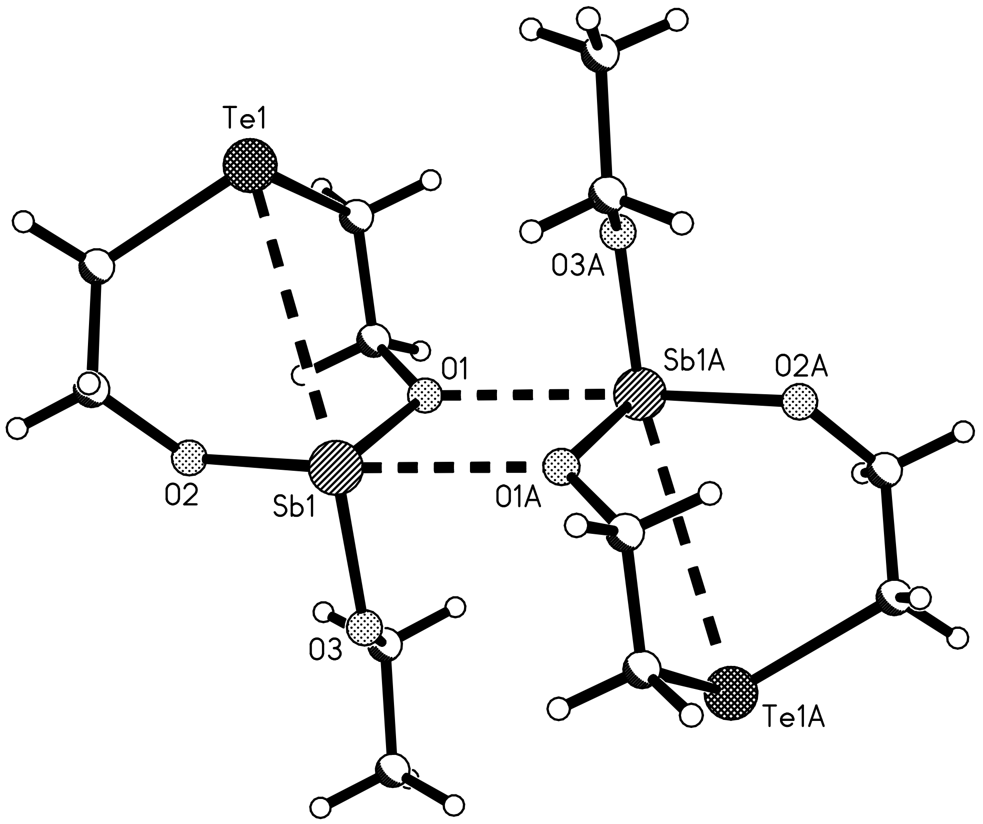

Figure S12: Dimer of **4** in the crystal structure. (The symmetry equivalent molecule is generated by symmetry operation -x+3/2, -y+1/2, -z+1/2.)

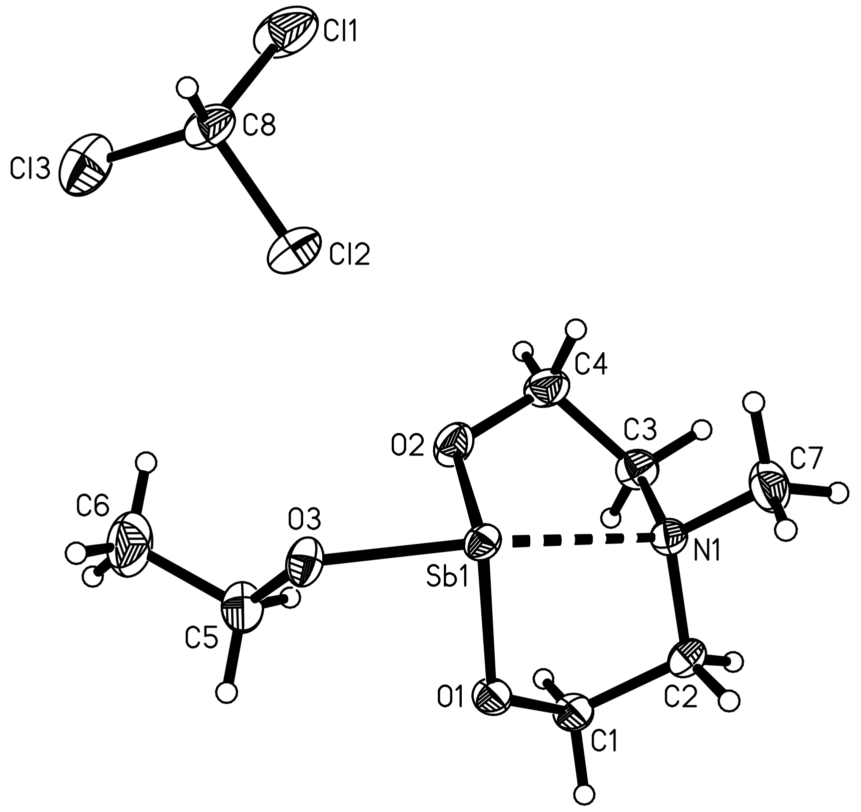

Figure S13: Asymmetric unit of **6**·$CHCl_3$ with atomic numbering scheme, shown with 50 % probability ellipsoids.

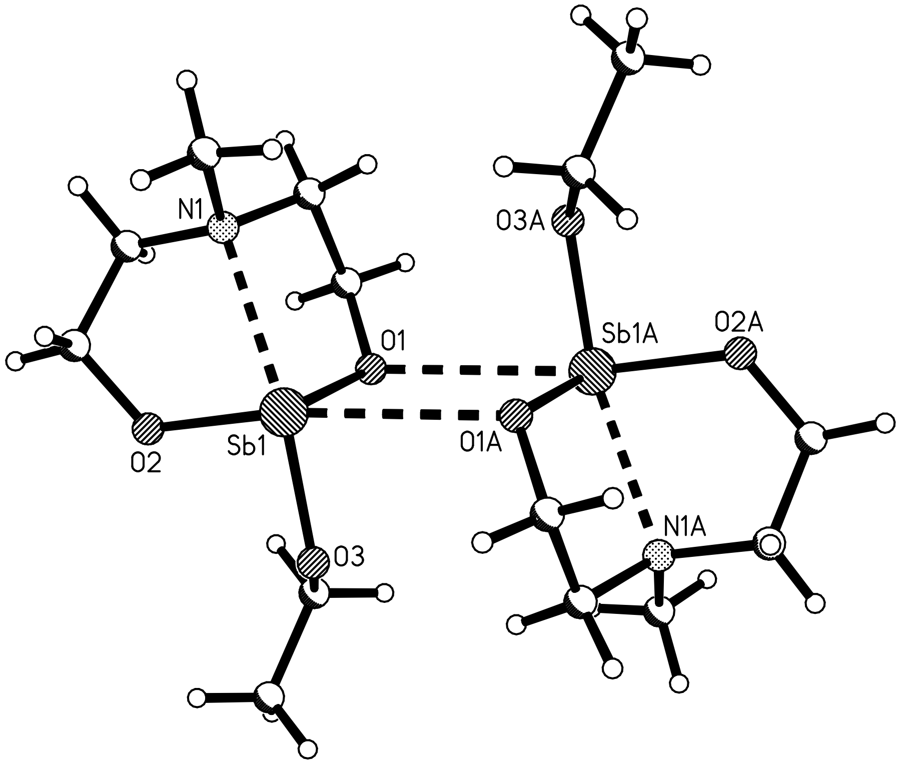

Figure S14: Dimer of **6** in the crystal structure. Chloroform molecules are omitted for clarity. (The symmetry equivalent molecule is generated by symmetry operation 1-x, 2-y, 1-z.)

7. Additional data from AIM analysis

One table is shown below for each compound.
Parameters are: charge density (ρ), its Laplacian ($\nabla^2\rho$), kinetic energy density (G), total energy density (H), and electronic potential energy density (V).
All values are given in atomic units.

Compound **1**	**ρ**	**$\nabla^2\rho$**	**G**	**G/ρ**	**H**	**V**
Bond critical points						
Sb1-O10	0.028	0.187	0.039	1.395	0.008	−0.031
Sb1-O2	0.206	0.779	0.253	1.230	−0.058	−0.311
Sb1-O3	0.206	0.797	0.257	1.249	−0.058	−0.315
Sb1-O17	0.226	0.806	0.273	1.209	−0.072	−0.345
Ring critical points						
Sb1-O2-C4-C7-O10	0.016	0.106	0.022	1.402	0.005	−0.017
Sb1-O3-C14-C11-O10	0.015	0.100	0.020	1.355	0.005	−0.015

Compound **2**	**ρ**	**$\nabla^2\rho$**	**G**	**G/ρ**	**H**	**V**
Bond critical points						
Sb1-S2	0.022	0.110	0.022	1.017	0.005	−0.017
Sb1-O3	0.204	0.804	0.258	1.266	−0.057	−0.314
Sb1-O4	0.208	0.796	0.259	1.247	−0.060	−0.319
Sb1-O17	0.223	0.777	0.264	1.187	−0.070	−0.334
Ring critical points						
Sb1-O3-C5-C8-S2	0.014	0.068	0.016	1.154	0.001	−0.014
Sb1-O4-C14-C11-S2	0.013	0.066	0.014	1.081	0.003	−0.011

Compound **4**	**ρ**	**∇^2ρ**	**G**	**G/ρ**	**H**	**V**
Bond critical points						
Sb2-Te1	0.008	0.048	0.009	1.135	0.003	−0.006
Sb2-O3	0.208	0.813	0.263	1.263	−0.059	−0.322
Sb2-O4	0.204	0.828	0.263	1.289	−0.055	−0.318
Sb2-O5	0.221	0.798	0.269	1.217	−0.070	−0.339
Ring critical points						
Sb2-O3-C6-C9-Te1	0.006	0.027	0.006	1.076	0.001	−0.005
Sb2-O4-C15-C12-Te1	0.006	0.022	0.006	1.032	−0.001	−0.007

Compound **5**	**ρ**	**∇^2ρ**	**G**	**G/ρ**	**H**	**V**
Bond critical points						
Sb1-N16	0.040	0.202	0.043	1.072	0.007	−0.036
Sb1-O2	0.204	0.793	0.256	1.252	−0.057	−0.313
Sb1-O3	0.203	0.779	0.252	1.241	−0.057	−0.309
Sb1-O21	0.220	0.775	0.262	1.190	−0.068	−0.330
Ring critical points						
Sb1-O2-C4-C5-N16	0.018	0.103	0.022	1.190	0.004	−0.018
Sb1-O3-C13-C10-N16	0.019	0.106	0.023	1.225	0.003	−0.020

Compound **6**	**ρ**	**∇^2ρ**	**G**	**G/ρ**	**H**	**V**
Bond critical points						
Sb1-N16	0.044	0.210	0.045	1.037	0.007	−0.038
Sb1-O2	0.203	0.795	0.255	1.257	−0.057	−0.312
Sb1-O3	0.203	0.781	0.253	1.244	−0.057	−0.310
Sb1-O21	0.221	0.779	0.263	1.194	−0.069	−0.332
Ring critical points						
Sb1-O2-C4-C5-N16	0.019	0.103	0.022	1.177	0.004	−0.018
Sb1-O3-C13-C10-N16	0.019	0.107	0.023	1.212	0.003	−0.020

8. Coordinates of optimized molecules

8.1 B97-3c Optimization

Triethoxyantimony

Sb	-2.63137770931437	0.69041978652927	1.33513632032850
O	-4.24800048295930	0.84891764242243	0.19445169521205
H	-4.99416926315764	4.10099048119006	-0.38268095130873
H	-5.53731476596036	3.08384108599079	0.95677377132128
H	-3.83444843926426	3.53624370415310	0.81021754228966
C	0.67384232482319	0.20908828180563	-0.15990255567735
H	0.28768472485676	2.28649501894956	-0.57046965907179
H	0.12848996080876	1.84881318914324	1.12083669593358
O	-1.46928553455157	1.37116379008584	-0.14345460132950
H	-5.35884008744630	1.79504092060320	-1.19496139020133
C	-3.92869220017250	-2.55006655694517	-0.33056768150277
O	-2.44334573943259	-1.25922703316382	1.08717731546947
H	-2.48958213066981	-1.09716039588734	-0.99472736393928
H	0.35305260662353	-0.57247334805588	0.52758000392782
C	-2.59706243177735	-1.84932479857949	-0.20972458696431
C	-0.07051256560681	1.50026194993023	0.09812636687481
H	-1.77372309459440	-2.55831913694486	-0.32804554458350
H	-4.00897162982732	-3.04884707686520	-1.29788639118826
H	-4.04336875618120	-3.30059414088191	0.45004829265273
H	-4.74092643206403	-1.83243877238449	-0.24434924446622
C	-4.47823260540065	2.01904326174413	-0.58904977723692
H	0.48792853090712	-0.13921716623191	-1.17429831694641
H	1.74818890396857	0.35275879225440	-0.03689642632904
C	-4.72733577250756	3.25313703778454	0.24915697265956
H	-3.64273041109384	2.18617748334759	-1.27120348592024

2-ethoxy-1,3,6,2-trioxastibocane (**1**)

Sb	3.29296168739100	2.03449764614050	2.96359145275668
O	2.32388328828122	2.83037462467968	1.37889655855433
O	5.05081603623005	3.00169324175807	2.77033576185036
C	2.25423014684986	2.00379190777037	0.23952262540252
H	1.60756326291255	2.48821851266101	-0.49681406063262
H	1.80441303427395	1.02877679635001	0.46953383364343
C	3.61988051051716	1.78660919002167	-0.37099603099805
H	3.56255116418155	1.11612391540692	-1.23279480201619
H	4.02901138185967	2.74221411612684	-0.69932821238991
O	4.46400289017580	1.19317538032647	0.62370247832795
C	5.74785793826150	1.81023361276332	0.77265534433939
H	6.22400360661008	1.93582644928384	-0.20427293835628
H	6.33705428097323	1.10657261075204	1.35844244439206
C	5.64990567067133	3.13186753559543	1.50388584178809
H	6.66333169387161	3.51800813461773	1.64885742231990
H	5.11103024386628	3.86396430932273	0.89156831791040
O	2.58896630720929	3.37022681971697	4.23505411753613
C	2.62893824709875	4.77069506411199	3.94917863765111
H	1.88410221679428	5.00713884325464	3.18467064844812
H	3.60847924216386	5.04516054175909	3.55017099740773
C	2.34635302036311	5.53465504186206	5.21970883227482
H	2.34597465742801	6.60791631185066	5.02471159887197

H	3.10289015139046	5.32321149826150	5.97356703777816
H	1.37447332062528	5.26056089560639	5.62698009313984

2-ethoxy-1,3,6,2-dioxathiastibocane (**2**)

Sb	-0.05194758431352	3.60087359620704	4.55860590933203
S	1.85266428238906	6.07456485299547	4.77675246491731
O	-0.47417817452698	4.55982421806587	6.28870230877026
O	1.75258731116975	2.90546612052930	5.12416820316791
C	-0.46543002026217	5.96409283119480	6.30291214083747
H	-0.96415450387637	6.28483407120954	7.22302573341996
H	-1.04145908574187	6.38786475636172	5.46999637121359
C	0.93368879514247	6.55535606462214	6.28816612240906
H	1.50224373677460	6.22118839706115	7.15243156731552
H	0.88621989948971	7.64300563044982	6.31195236928595
C	3.10367564108211	4.91063814127071	5.43278289918159
H	3.71750638950200	4.66560490955820	4.56801374855172
H	3.72743704560595	5.43758637633759	6.15493335991773
C	2.52299735289047	3.64680310430599	6.03479145108984
H	3.35973609166531	3.01204043462298	6.34454117753647
H	1.95267933843197	3.88813025552622	6.93667407976727
O	-0.99585632024066	1.95162370710690	5.12330218961883
C	-0.77437840256722	1.35333911117238	6.40034414272721
H	-1.23791859069629	1.96769801727131	7.17723891223487
H	0.29688272358416	1.30728616837442	6.61374731717505
C	-1.36782947522554	-0.03482655585950	6.39992302567844
H	-2.43345669975759	0.00155997137941	6.17922357741202
H	-1.23600609503562	-0.50545157229293	7.37527574578071
H	-0.88697465548374	-0.66011260747061	5.64946018265902

2-ethoxy-1,3,6,2-dioxaselenastibocane (**3**)

Sb	0.10548356559811	3.59536972503130	4.66438372429627
Se	2.08621199015546	6.13628176599576	4.83902255647276
O	-0.29259237455606	4.54047308159541	6.40843954810068
O	1.91080867410560	2.88350112475170	5.20784709694099
C	-0.31715750473993	5.94439442154526	6.44515311034799
H	-0.83727778244828	6.23288313746825	7.36510786209123
H	-0.89576609173883	6.36924436288559	5.61459496218779
C	1.06223834279374	6.57299708156793	6.46514299169140
H	1.64344846666124	6.22275377904039	7.31373861543062
H	0.99563470434247	7.65782973881244	6.51543773319315
C	3.34616817448506	4.81834252278637	5.58427509211311
H	3.97957056081630	4.56030852935940	4.73839919057010
H	3.95354377400307	5.32347649078211	6.33331854776584
C	2.67933996617783	3.58454132696236	6.15172488968897
H	3.47140738407176	2.90647370675658	6.48867364212572
H	2.08844544438121	3.84869727367267	7.03322335785053
O	-0.84951100370702	1.94964021063265	5.22943523203994
C	-0.61145148638260	1.33424676763104	6.49502317585136
H	-0.92890814049570	2.00550702438853	7.29775245382053
H	0.45838331844683	1.14988948032525	6.62562855218836
C	-1.37900897115678	0.03576714479284	6.55864140602696
H	-1.22190949166662	-0.45256221153818	7.52148502283620
H	-1.05337656818833	-0.64365304620376	5.77257882252714

H	-2.44637395095855	0.21157056095801	6.43430841384219

2-ethoxy-1,3,6,2-dioxatellurastibocane (**4**)

Te	5.83660214077931	5.85232994383883	1.44816097413832
Sb	8.86478310087296	4.83095308641457	2.63790316294952
O	7.46501598589935	4.01734429174257	3.83693578857718
O	8.59955580492608	6.73164333326070	3.27314822783857
O	10.34035737682504	4.50382706396955	3.92694585455279
C	6.39815814852192	4.79772299072548	4.31621712930663
H	6.73635057510595	5.78488474223741	4.64147987704928
H	6.00675979838420	4.28612564930358	5.20370488372401
C	5.25908014433164	4.93517400183703	3.33189762414623
H	4.44415166408959	5.52802914641998	3.74157292480259
H	4.87306190065256	3.96175293892368	3.03792359853813
C	6.58538968499677	7.70903013479235	2.29109254800207
H	6.23315374465804	8.50822440122619	1.64261896738680
H	6.11048857409951	7.81542137952294	3.26263527889913
C	8.09506315497410	7.71502491718133	2.40449766446165
H	8.53809789122620	7.64926244315588	1.40255593772380
H	8.40236326937989	8.68414621859793	2.81469981158183
C	10.22212062244492	4.83440206291746	5.30971240183704
H	9.28360422712974	4.44173969286539	5.71077829014961
H	10.20004515355047	5.92138910656137	5.42778423279558
C	11.39557875898783	4.24595052142480	6.05580170834280
H	11.40797861025869	3.16137623595663	5.96098760873911
H	11.33761134726844	4.49763533438333	7.11573410706950
H	12.33520632063667	4.63160936274091	5.66340639738776

2-ethoxy-1,3,6,2-dioxazastibocane (**5**)

Sb	2.99072283287397	6.37071116063885	4.15428894443994
O	1.57632484476557	6.85188650583335	5.52023045046698
O	3.05417476542631	4.35242193551801	4.36131376546390
C	1.65235761277880	6.33240170815268	6.82052588211820
H	1.33764116625559	5.28148345644256	6.84000117559316
H	0.95113533661656	6.88574777177892	7.45145801914430
C	3.04380822449949	6.46489503946466	7.41585948000315
H	3.29723659224863	7.52235970115883	7.48428690385104
H	3.06501589540569	6.04373530496093	8.42830793384749
C	4.06565279123714	4.36714100937840	6.55017723025390
H	3.15708735354754	3.99697213954339	7.01998315500906
H	4.91337284786760	3.99814729205496	7.13443478369978
C	4.12067801524565	3.84882455196710	5.12198759180154
H	5.09537879072473	4.10611980911821	4.68079187721750
H	4.05435721929322	2.75747026688049	5.12719058724686
N	4.01700265835213	5.83082322559857	6.52885652495070
H	-0.09318558852060	5.64907892254665	0.79231332615507
C	-0.53349877695987	5.81401408394879	1.77465692087629
H	-1.43640221621348	5.20624289663593	1.85210710169359
H	-0.81849356026869	6.86253505788573	1.84679840735727
O	1.61893941570554	6.24252182957780	2.72162128425548
C	0.44329518283985	5.44976369276378	2.86747396074243
H	-0.00788746098597	5.62004872009761	3.84880153415200
H	0.70517931035821	4.38940964417667	2.80782644940768

H	4.93690474690620	6.22933327387677	6.64593571025230

2-ethoxy-6-methyl-1,3,6,2-dioxazastibocane (**6**)

Sb	2.99861768364141	6.32960033843298	4.14241798973022
O	1.55339012821547	6.78301905582203	5.48726190823781
O	3.07590081254441	4.30994458263378	4.32988596858922
C	1.64997350963615	6.30939025459640	6.80416287274593
H	1.33615882523090	5.25989769452510	6.86485184552915
H	0.95612992438536	6.88175694685634	7.42646943239867
C	3.04718183064300	6.47331176132193	7.37516609239353
H	3.28486838038705	7.53597916248263	7.40711143770762
H	3.09520426582523	6.08876474934451	8.40253781301881
C	4.00668273948042	4.36167782797901	6.55242935014664
H	3.06174708936729	4.03784318353433	6.98041926776416
H	4.80945247162535	3.97448617650688	7.18980548061920
C	4.10494914650576	3.80662869500379	5.14170916032596
H	5.09808973476801	4.02710831851511	4.72429472222319
H	4.01483125422391	2.71762751494413	5.17651337954148
N	4.02976799244232	5.82630867496324	6.50713450712888
C	5.37176529430346	6.37477986995080	6.63028686764478
H	5.35812598150524	7.43839377575946	6.40217967806171
H	6.03549293932037	5.88997105770930	5.91841364140880
H	5.78552054586734	6.23654819521039	7.63573763772642
O	1.64718075293560	6.20357086791842	2.69287763327003
C	0.48079359898281	5.39226483496300	2.80841287587072
H	0.08596651101389	5.43501297882767	3.82691585751144
H	0.74046281943613	4.34978884077382	2.60213706236125
C	-0.55890039596898	5.88174516412643	1.82830954706015
H	-0.84779067901385	6.90706852032062	2.05414846057002
H	-1.45106888729720	5.25503558029750	1.87333190555741
H	-0.17287127000717	5.85435837667970	0.81027560485614

2-ethoxy-1,4-oxathiastibpentan (**7**)

Sb	-2.80189848485732	-0.43536999171689	-1.31564560357948
O	-1.44537770621312	1.00150667050792	-1.01953191629222
O	-3.88354686442890	-0.17150650825474	0.34585674376273
C	-5.24477062319909	0.22968439283622	0.20100492713672
H	-5.47463360452321	0.86171771193503	1.06176286163993
H	-7.21674148690702	-0.64422284340614	0.13129258627859
H	-5.98405816325136	-1.57138445581931	-0.72643626658097
H	-6.02556507647333	-1.58921189474831	1.03537906761602
C	-0.58343249950142	0.72384219499182	0.07554649932330
H	-5.39320247596485	0.85547595424888	-0.68881187799045
C	-6.17361475416433	-0.96214447641028	0.15841404615782
C	0.06101396796107	-0.63696575579659	-0.06390509193636
H	0.19187170264455	1.49405011070853	0.07371257911295
H	-1.12917775764364	0.78399444369138	1.02127998683586
S	-1.21558427431049	-1.95845327679806	-0.26057375094343
H	0.73052952593294	-0.66304880432210	-0.92036084639327
H	0.62429557489928	-0.88392347164636	0.83434405585232

Dimer of **3**

Sb	0.12097991919334	3.56866437713873	4.56649652430102
Se	2.16418831028979	6.03272851074853	4.78124131443994
O	-0.19702227430274	4.46233363152995	6.38442074664980
O	1.93308301029510	2.82907453643446	5.21653608217707
C	-0.25191215271701	5.86270960619727	6.38967073645025
H	-0.77206186754869	6.17387879677415	7.30312586645888
H	-0.84252113487244	6.25927142855630	5.55090076634494
C	1.11504889183531	6.52322526104842	6.37866917929147
H	1.70359887363004	6.23134147129604	7.24446334601155
H	1.02355989447094	7.60767573167178	6.37506663176701
C	3.39831444130396	4.74345058687127	5.59914835999515
H	4.05488571919065	4.46633698724900	4.77979784691859
H	3.96904355372119	5.26387000282645	6.36601278988916
C	2.70670997089633	3.52596551221513	6.16735710336834
H	3.47893504387732	2.83317289712543	6.52102125113976
H	2.08848983766086	3.79083626295914	7.02545509856219
O	-0.79950975269085	1.88903614096903	5.15262462219159
C	-0.64805086067001	1.37446724227825	6.47115220466574
H	-1.12964613635418	2.04167293691023	7.19084981487029
H	0.41234625017603	1.32432596651381	6.73606238678300
C	-1.26349614316541	-0.00355126012693	6.53510762290091
H	-1.18918345688980	-0.40821407197019	7.54534860946475
H	-0.75635982124464	-0.68984624064181	5.85769241203223
H	-2.31625732631081	0.03117174009227	6.25823018790584
Sb	3.04103976631864	1.43297923279458	3.43197132444712
Se	0.99782983847274	-1.03108388240455	3.21722658809987
O	3.35904173164999	0.53930947614715	1.61404727364477
O	1.22893697444465	2.17256988533081	2.78193147163595
C	3.41393078741750	-0.86106653743187	1.60879762409300
H	3.93408053853277	-1.17223627211995	0.69534267852949
H	4.00453935867560	-1.25762852396987	2.44756781316343
C	2.04696935875924	-1.52158138722910	1.61979901164304
H	1.45841971673895	-1.22969725825455	0.75400472659665
H	2.13845771229333	-2.60603191178291	1.62340164624356
C	-0.23629544161386	0.25819451527998	2.39931898790257
H	-0.89286685729348	0.53530839677115	3.21866928186732
H	-0.80702454338329	-0.26222472738544	1.63245442891200
C	0.45530984024494	1.47567917942765	1.83111033615577
H	-0.31691474003855	2.16847220752981	1.47744594051795
H	1.07353000411783	1.21080797624331	0.97301250196141
O	3.96153036933300	3.11260692347143	2.84584310972237
C	3.81007060944995	3.62717669100924	1.52731596363821
H	4.29166534372922	2.95997137211110	0.80761763706785
H	2.74967332134375	3.67731827001049	1.26240654943960
C	4.42551604249252	5.00519513494052	1.46336092877090
H	4.35120310441914	5.40985833190623	0.45312010587904
H	3.91838005986718	5.69148994858587	2.14077655847427
H	5.47827731425384	4.97047190435200	1.74023800701405

8.2 PBE0/def2-TZVPP Optimization

2-ethoxy-1,3,6,2-trioxastibocane (**1**)

Sb	3.23569382790804	2.02513583710860	2.96028007250329
O	2.26995381822923	2.72965085801111	1.39843064002905
O	4.90630849390497	3.03557201271501	2.76365166142300
C	2.26234036637366	1.97526059364354	0.22854719244962
H	1.62883899324165	2.47109212847032	-0.50291281735921
H	1.84447998932231	0.97955380412973	0.39326115320084
C	3.64957232618653	1.83611911041956	-0.33236057536237
H	3.65392850530999	1.20921447352765	-1.22136326512870
H	4.03129632425145	2.81730265784894	-0.59792964563147
O	4.45625950758742	1.24644905543474	0.66551761897809
C	5.72273047197088	1.84239378291026	0.84326582389685
H	6.22583153380988	1.96773496267983	-0.11388092923959
H	6.29473624301220	1.14298377651308	1.44214301059918
C	5.60140752623968	3.15842160352043	1.56701374109505
H	6.60349192170038	3.52321775244156	1.78395329043854
H	5.12573499723387	3.89840801075135	0.92174066374210
O	2.49945163663538	3.35347151595545	4.14339290161905
C	2.52446936055240	4.73518457531088	3.86634028673656
H	1.66167639277282	4.99384520638418	3.25610757653398
H	3.41468345939622	4.99321207016602	3.29706768211917
C	2.50517956031037	5.49900694450047	5.15895807822281
H	2.51198531381896	6.56710139816070	4.96874520120055
H	3.37424402562477	5.25220705634511	5.75735674997291
H	1.61837940460667	5.25897381305124	5.73350188796055

2-ethoxy-1,3,6,2-dioxathiastibocane (**2**)

Sb	0.02869959795023	3.55128985895694	4.53314296329341
S	1.82168631697351	6.07871152505594	4.82321211546862
O	-0.45111069120182	4.47329988226230	6.20475984559391
O	1.80282383080365	2.96011439272146	5.12379733567191
C	-0.49633962564750	5.85929781554228	6.26992812988331
H	-1.02576270730382	6.13451058353551	7.18047393461314
H	-1.06072463079430	6.28890397911728	5.43903148787491
C	0.87853473856892	6.48776587634188	6.30249733211819
H	1.43428889209976	6.14142441832884	7.16520363138273
H	0.80045499084643	7.56709357730373	6.36254269503469
C	3.09060310001825	4.97461700696824	5.45859227647555
H	3.71356364364556	4.76245882990316	4.59672691933229
H	3.69344410003577	5.50730128106902	6.18643240867181
C	2.55268033959354	3.68856396659898	6.03669052596114
H	3.40152917281109	3.07491754062504	6.33424454961506
H	1.98113141144767	3.90050215885528	6.93972399189516
O	-0.82079285054137	1.90844670759536	5.09343317835227
C	-0.61816679472656	1.30799325573779	6.35007818710380
H	-0.86312282520493	2.01055258733789	7.14446571573226
H	0.42962131819833	1.03748765624351	6.46682772730213
C	-1.48773077437193	0.08766146478122	6.45061945776619
H	-2.53266156562900	0.35691447878496	6.35143562859320
H	-1.34923741040710	-0.40302429900505	7.40814453312454
H	-1.24468257716440	-0.61781454466172	5.66496042913949

2-ethoxy-1,3,6,2-dioxaselenastibocane (**3**)

Sb	0.11596644201748	3.57310840942768	4.63833637636993
Se	2.08096858110543	6.13541680868374	4.88856823651312
O	-0.29701586342025	4.50641712559817	6.31946171588498
O	1.88912091238387	2.93148116506805	5.17692018791068
C	-0.33153278789884	5.89140491508970	6.41203526331769
H	-0.87440265427552	6.14627760435757	7.32152484916330
H	-0.88340215386081	6.34274826389239	5.58462052911399
C	1.04126140703065	6.51343333229178	6.48554074643223
H	1.59840723128093	6.12830918723425	7.32994705838586
H	0.97468124570043	7.58982786416809	6.57996801410407
C	3.32219431734114	4.82611628097972	5.60528721391631
H	3.96180621019465	4.58625563100351	4.76448788059415
H	3.92105250590247	5.30875590436803	6.36807905838727
C	2.65371285470528	3.58419146318051	6.13437786177711
H	3.43583025584891	2.89677691303782	6.45572297665759
H	2.06444520684566	3.82750282227451	7.01740241741503
O	-0.76869916364371	1.95881920339685	5.23070310467891
C	-0.53356452015797	1.35431318886556	6.47992574057749
H	-0.77409430918863	2.04960002994529	7.28207646836887
H	0.52017665076291	1.09860565502133	6.57646371286277
C	-1.38090665566396	0.11934184030567	6.59214723268771
H	-1.21753531140415	-0.37019890102921	7.54642673272071
H	-1.13922187790585	-0.58101303390597	5.80153631336711
H	-2.43189752370017	0.37048232674483	6.51177630879286

2-ethoxy-1,3,6,2-dioxatellurastibocane (**4**)

Te	5.81925578698147	5.87704187484953	1.51177000714659
Sb	8.90113506239684	4.83544485947428	2.61601488812921
O	7.50725440832927	4.03587935375742	3.73553746973610
O	8.61703690029153	6.68538926650037	3.21441121972015
O	10.29974590295295	4.54949474914339	3.92270682206832
C	6.47276050638602	4.77374466897451	4.29979203488030
H	6.81833500973770	5.74296779294702	4.65438718227940
H	6.12045033784465	4.22454053509576	5.17339100957790
C	5.30372984871016	4.95660501183233	3.36667241067620
H	4.52099477379003	5.54906759670773	3.82333907298334
H	4.88731468201455	3.99972092444537	3.07569939417755
C	6.60134434454853	7.68664298460339	2.32858386945070
H	6.23683490234622	8.49577480714964	1.70865333640638
H	6.16939091028715	7.79175107273832	3.31580129684019
C	8.10820934202496	7.67952107768166	2.38746314430499
H	8.50845573226431	7.61262889624440	1.37360828571971
H	8.44339631527317	8.63572412050227	2.78894394125239
C	10.15896556457945	4.86108824092932	5.28822839945138
H	9.22018016769397	4.46246473283519	5.66987711384585
H	10.13002840517467	5.94125577643415	5.41858343193316
C	11.31212152420762	4.27386455693106	6.05106885048973
H	11.33017132703871	3.19579383168334	5.94402573406054
H	11.23085656669355	4.51223393051117	7.10643029167947
H	12.25260967843227	4.66635833802822	5.68320579319032

2-ethoxy-1,3,6,2-dioxazastibocane (**5**)

Sb	2.97625998976806	6.36467260843058	4.14884694681009
O	1.61985646633174	6.82381521501669	5.49815669895228
O	3.03067135300709	4.40659408715956	4.38609400326628
C	1.66502440821440	6.31719289459850	6.78829542281888
H	1.35651787169928	5.27009250481825	6.80366261646802
H	0.95360475277648	6.86494856760961	7.40226426570572
C	3.03967758894907	6.45276013282742	7.40009759474052
H	3.28467012715467	7.50637105297099	7.47902572053951
H	3.04783569797958	6.02854355996632	8.40521941449825
C	4.05732732508783	4.38141685673407	6.53811598897160
H	3.15696118420637	4.01327069177911	7.01598099767584
H	4.90554544759771	4.00263261554779	7.10401780519026
C	4.08762098704071	3.87759356421118	5.11393261836203
H	5.05387773434981	4.12858903584208	4.66519895704820
H	4.01023960791273	2.79294297059465	5.10495462968856
N	4.01505723661620	5.83154253036376	6.52927769851097
H	-0.04947095498967	5.62909847947542	0.83198783738950
C	-0.50263141678557	5.80792296434626	1.80008052571602
H	-1.41017308937627	5.21726778609709	1.86862831997770
H	-0.77057242105607	6.85639926946471	1.85850887114610
O	1.61935614068619	6.21445679410472	2.77354075391623
C	0.45003257769349	5.44542990114091	2.90375531070046
H	-0.01444322251817	5.62521340232945	3.87171405078797
H	0.69829115013116	4.38662877870230	2.85568221622596
H	4.92566145752293	6.22869273586815	6.65018973489261

2-ethoxy-6-methyl-1,3,6,2-dioxazastibocane (**6**)

Sb	2.97130426242263	6.34358305841476	4.15127863299261
O	1.58241727481972	6.74411669369403	5.48871007105988
O	3.06439528750782	4.38121753991472	4.33311335929255
C	1.66607836607892	6.30245901343356	6.80058049726945
H	1.34996281173785	5.26041636497743	6.87656239433661
H	0.97623261709548	6.88259275037277	7.40978324592980
C	3.05672924804131	6.46664343908812	7.36588978943183
H	3.29437565312130	7.52489723754392	7.39816057826654
H	3.10624287422232	6.08414234913157	8.38818463844611
C	3.98943088778651	4.37644809016189	6.52796879850278
H	3.04565503508608	4.05461046042638	6.94940119326862
H	4.78602697056113	3.97598810446634	7.15539484125830
C	4.08033519115356	3.84953872558015	5.11582356412336
H	5.06809090139764	4.07474662322925	4.70289569883751
H	3.98658047664205	2.76629322410016	5.12400517029156
N	4.02325101130916	5.82652396123627	6.50047582844979
C	5.35771178702883	6.35441631508563	6.63025136477555
H	5.35749066773039	7.41784013315094	6.42347314305154
H	6.01556751558689	5.87719798907985	5.91441760262244
H	5.76694455091604	6.19337789953680	7.62878954606040
O	1.63007073019074	6.19808131528118	2.76209521516464
C	0.47763989733583	5.39878084810594	2.85741886771329
H	0.04683557751995	5.47091851523082	3.85430292361616
H	0.74123464222170	4.35546914847015	2.69340351240986
C	-0.52149269945492	5.85305370912707	1.83121126313862
H	-0.80799513401619	6.88273565913626	2.01034027139784

H	-1.41394024300156	5.23697304027924	1.86624418615944
H	-0.09955316104153	5.78882079174400	0.83502180213217

Dimer of **3**

Sb	0.14821612530485	3.61043985060421	4.52825624332241
Se	2.19632009077797	6.11284975803878	4.86353195024956
O	-0.18313404063166	4.48275859541673	6.28404447896990
O	1.92201331236018	2.92912623004446	5.15696693837024
C	-0.23773642551610	5.86535695147390	6.36507668223366
H	-0.79129159647324	6.12989724275936	7.26615044847574
H	-0.78680259891214	6.30499590136817	5.52743250187997
C	1.12730013882782	6.50792755753940	6.43876601539274
H	1.68093236354179	6.15065937455846	7.29782089965227
H	1.04571370741730	7.58533430740019	6.50889671341863
C	3.39553653279245	4.78854192242896	5.61185097184543
H	4.04066939463877	4.52467574088905	4.78313395701506
H	3.98203338188205	5.25997515000949	6.39079043040950
C	2.68647940577512	3.56723496631969	6.13163490602558
H	3.44169169508016	2.85675241219957	6.46951503727716
H	2.06992408678000	3.81325838169361	6.99146648519842
O	-0.73999233493433	1.98330751041750	5.12648538046394
C	-0.59935259748213	1.46764306053819	6.42652966889550
H	-1.12564269220350	2.10114211103293	7.13723085522259
H	0.44957852384979	1.46204140824905	6.71987499531387
C	-1.14678849935972	0.06886557747605	6.46720274285054
H	-1.08243414363592	-0.33689353912403	7.47121615585563
H	-0.58990485711900	-0.58366821277896	5.80398112327729
H	-2.18657547550033	0.05721622608422	6.16152100251911
Sb	3.01340671371589	1.39136477279269	3.47038913297737
Se	0.96532334486991	-1.11094508559790	3.13502165087425
O	3.34478919754189	0.51910910993448	1.71458124483846
O	1.23965819083942	2.07275310931388	2.84164756545096
C	3.39939198906764	-0.86348412726825	1.63350920057255
H	3.95295728004753	-1.12800444927249	0.73243539244357
H	3.94844364309264	-1.30314542636999	2.47115000411866
C	2.03434971005553	-1.50603861685362	1.55979345414768
H	1.48073330285160	-1.14875428125837	0.70073306268662
H	2.11592182934845	-2.58344751679831	1.48965784935604
C	-0.23386865165824	0.21337981391390	2.38669567495569
H	-0.87900648586006	0.47724771521666	3.21540674645642
H	-0.82036010815914	-0.25803670582985	1.60774309956843
C	0.47520965961781	1.43468219740684	1.86694606738462
H	-0.27998599508263	2.14518114044156	1.52906295846386
H	1.09178350119458	1.18866724042555	1.00712631847225
O	3.90169223854012	3.01848261548602	2.87223815068917
C	3.76233521139631	3.53339524214974	1.57176034206772
H	4.28964943707173	2.89970187384753	0.86198888413181
H	2.71373126632325	3.53847428256669	1.27722236430928
C	4.30933361220129	4.93234241732070	1.53098282001184
H	4.24600720515374	5.33754904849011	0.52667997119673
H	3.75145255385706	5.58503218794780	2.19321711108545
H	5.34876085671332	4.94451195735533	1.83786634960536

9. RMSD of experimental and optimized structures on non-hydrogen atoms

The geometries from the crystal structure data were compared with the optimized geometries. The root-mean-square deviation of atomic positions (RMSD) was applied for this purpose. This method provides a measure of the absolute differences between calculated values and experiment. (Literature: J. B. Foresman, AE. Frisch: Exploring Chemistry with Electronic Structure Methods, Gaussian Inc. Pittsburgh, Second Edition 1996, p. 145.)
The positions of all non-hydrogen atoms were included in the analysis. Bond lengths to hydrogen are too short, when obtained from X-ray structure data. Therefore, X-H bond lengths were not included in the analysis shown below.

Compound	B97-3c optimized structure [Å]	PBE0/def2-TZVPP optimized structure [Å]
**1**	0.176	0.115
**2**	0.197	0.142
**3**	0.168	0.132
**4**	0.168	0.139
**6**	0.338	0.317
**7**	0.185	0.233

10. ^{13}C NMR spectrum of the reaction product of 6 with formic acid

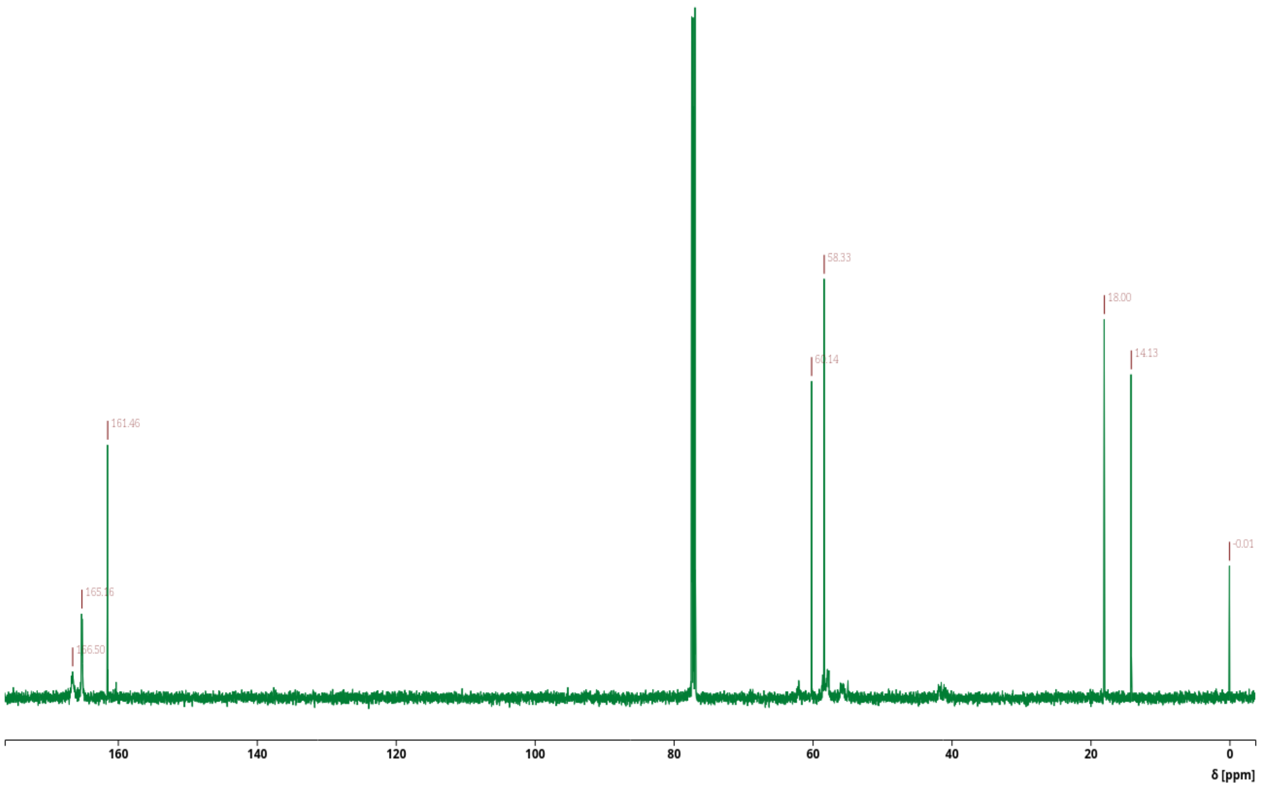

Figure S15: ^{13}C NMR spectrum of the reaction mixture of **6** with formic acid in $CDCl_3$.
